# Supplementary material for: Selective sorting of microRNAs into exosomes by phase-separated YBX1 condensates
Source: eLife. 2021 Nov 12;10:e71982. doi: 10.7554/eLife.71982 (PMC8612733; doi:10.7554/eLife.71982)
Supplement: Figure 3—figure supplement 2—source data 1. [file elife-71982-fig3-figsupp2-data1.zip › Figure 3-figure supplement 2-source data 1/Uncropped SDS-PAGE corresponding to Figure 3-figure supplement 2.pdf]

Figure 3- figure supplement 2D

uncropped gels

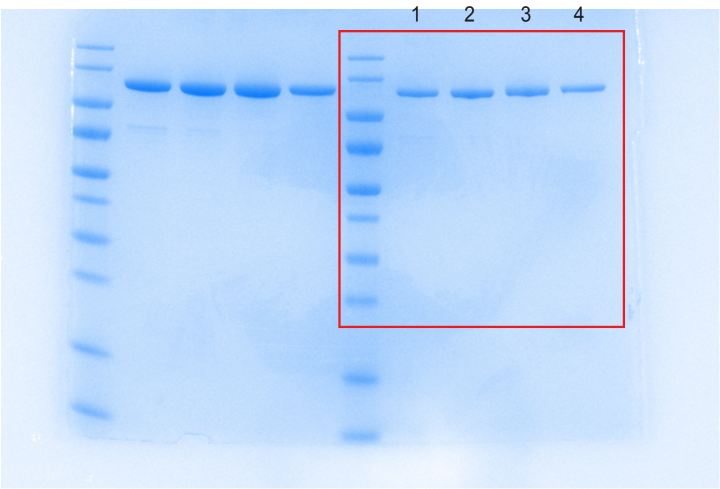

1. YBX1-WT
2. YBX1-F85A
3. YBX-CTD-Y to S
4. YBX1-CTD-RK to G

D

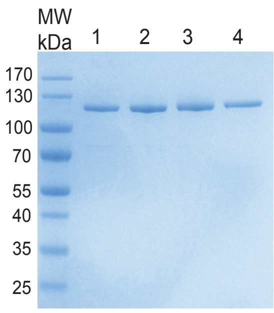

1. YBX1-WT
2. YBX1-F85A
3. YBX-CTD-Y to S
4. YBX1-CTD-RK to G

Figure 3- figure supplement 2D. SDS-PAGE of YBX1 wild-type and variants tagged with 6xHis-MBP-mGFP.
