# Supplementary figures and images for: Selective sorting of microRNAs into exosomes by phase-separated YBX1 condensates
Source: eLife. 2021 Nov 12;10:e71982. doi: 10.7554/eLife.71982 (PMC8612733; doi:10.7554/eLife.71982)

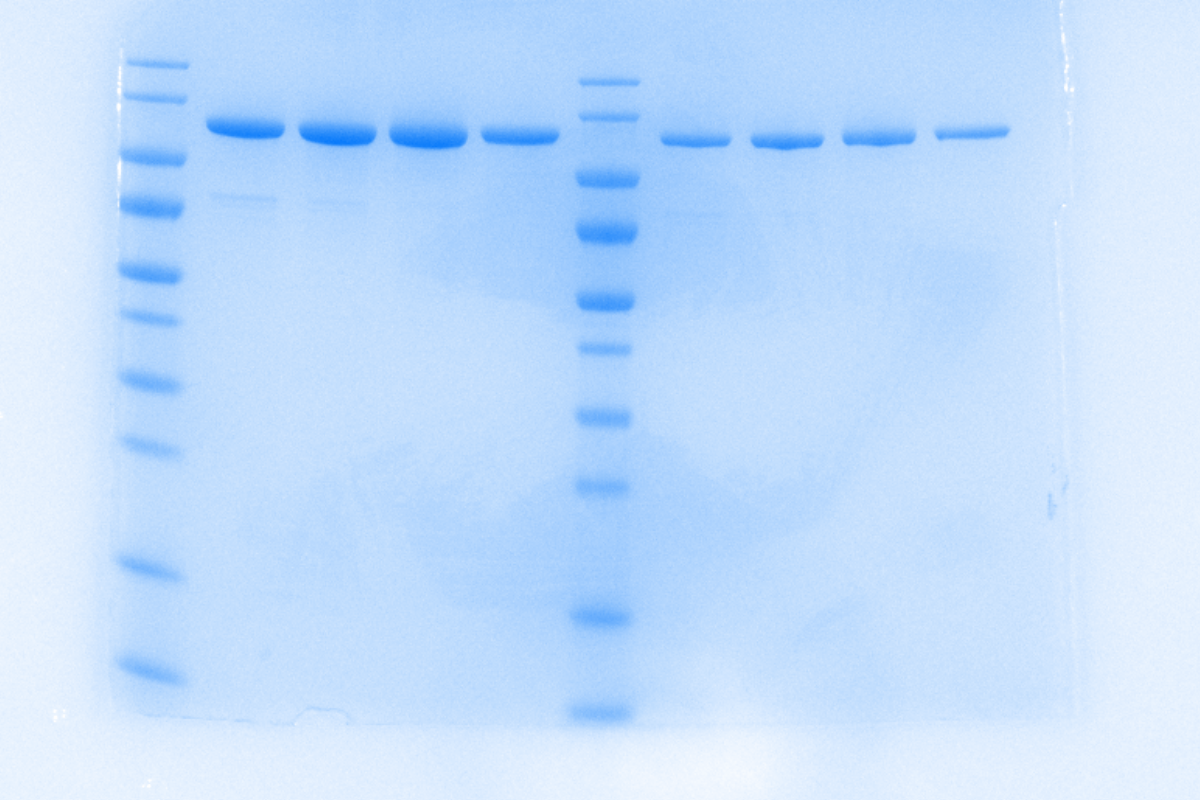

Supplement: Figure 3—figure supplement 2—source data 1. [file elife-71982-fig3-figsupp2-data1.zip › Figure 3-figure supplement 2-source data 1/uncropped SDS-PAGE corresponding to Figure 3-figure supplement 2.tif]

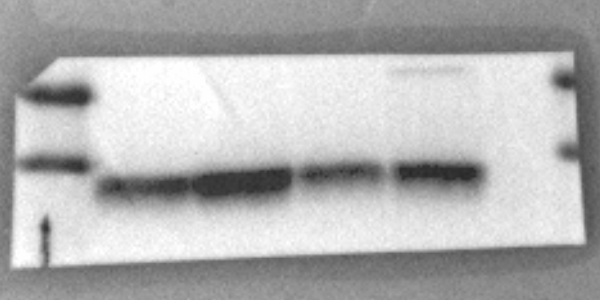

Supplement: Figure 4—source data 1. [file elife-71982-fig4-data1.zip › Figure 4-source data 1 for Figure 4C/Figure 4C-unmodified blot for CD9 in sedimentable particles.tif]

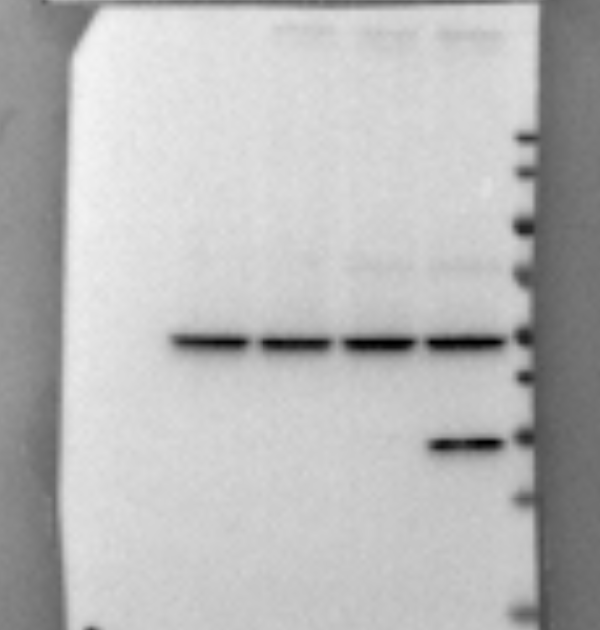

Supplement: Figure 4—source data 1. [file elife-71982-fig4-data1.zip › Figure 4-source data 1 for Figure 4C/Figure 4C-unmodified blot for DDX6 in cells.tif]

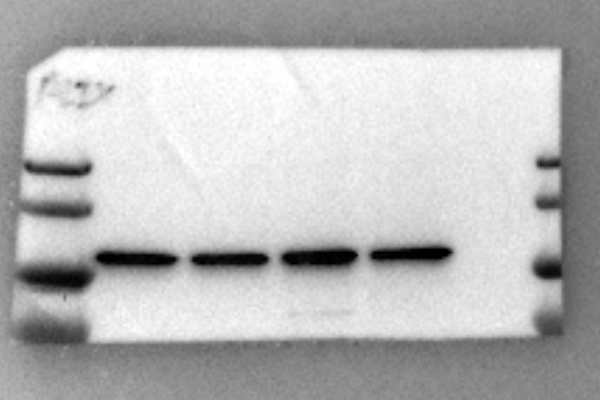

Supplement: Figure 4—source data 1. [file elife-71982-fig4-data1.zip › Figure 4-source data 1 for Figure 4C/Figure 4C-unmodified blot for ALIX in sedimentable particles.tif]

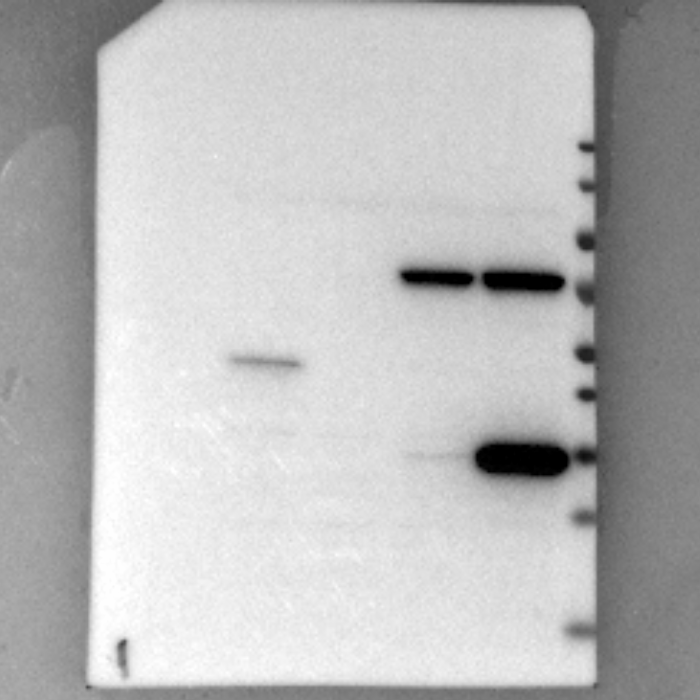

Supplement: Figure 4—source data 1. [file elife-71982-fig4-data1.zip › Figure 4-source data 1 for Figure 4C/Figure 4C-unmodified blot for YBX1 in cells.tif]

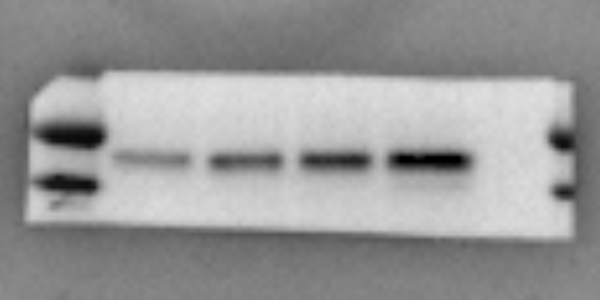

Supplement: Figure 4—source data 1. [file elife-71982-fig4-data1.zip › Figure 4-source data 1 for Figure 4C/Figure 4C-unmodified blot for Flotillin-2 in sedimentable particles.tif]

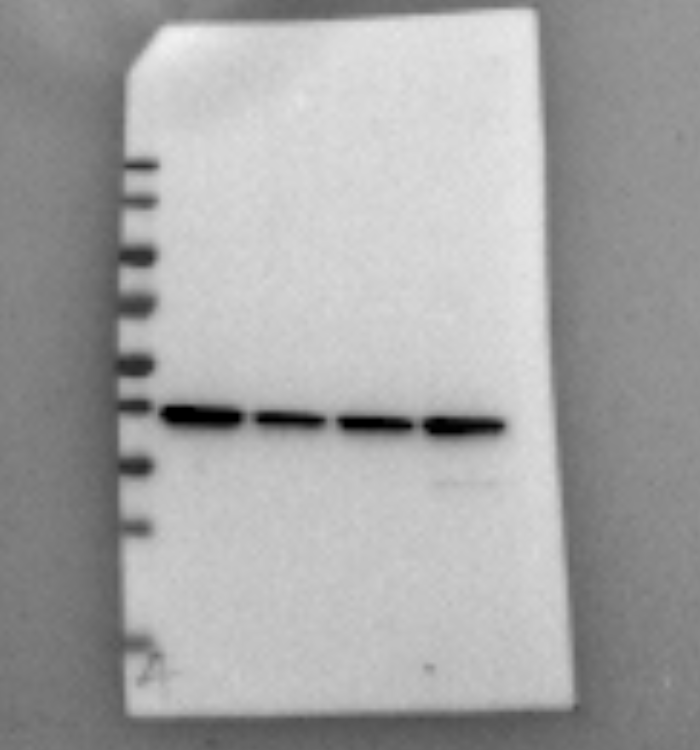

Supplement: Figure 4—source data 1. [file elife-71982-fig4-data1.zip › Figure 4-source data 1 for Figure 4C/Figure 4C-unmodified blot for Actin in cells.tif]

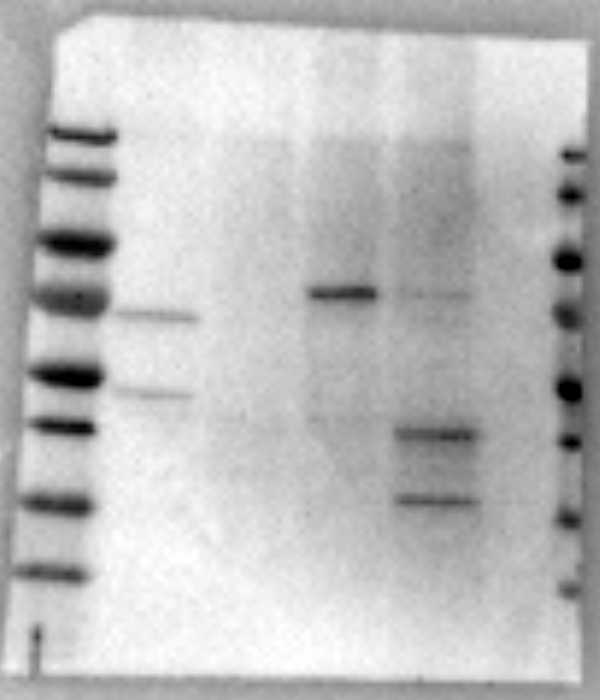

Supplement: Figure 4—source data 1. [file elife-71982-fig4-data1.zip › Figure 4-source data 1 for Figure 4C/Figure 4C-unmodified blot for YBX1 in sedimentable particles.tif]

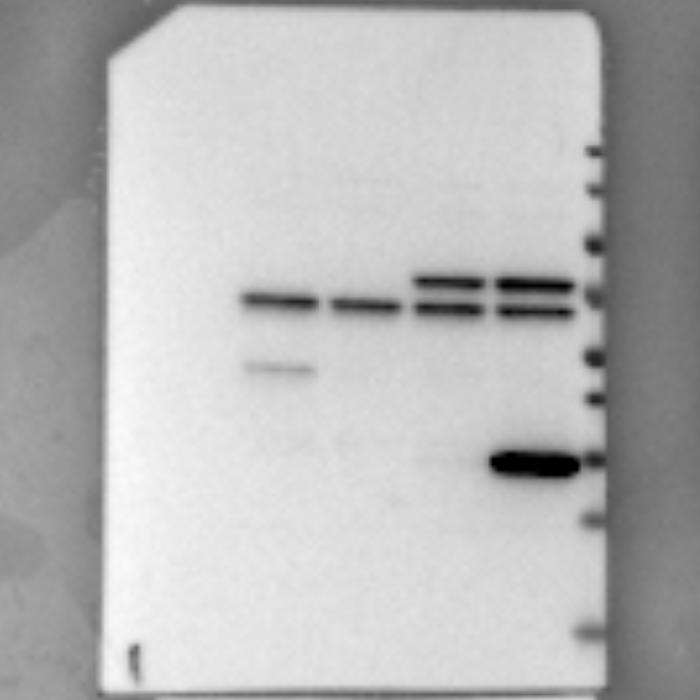

Supplement: Figure 4—source data 1. [file elife-71982-fig4-data1.zip › Figure 4-source data 1 for Figure 4C/Figure 4C-unmodified blot for G3BP1 in cells.tif]

Figure 4C uncropped blots

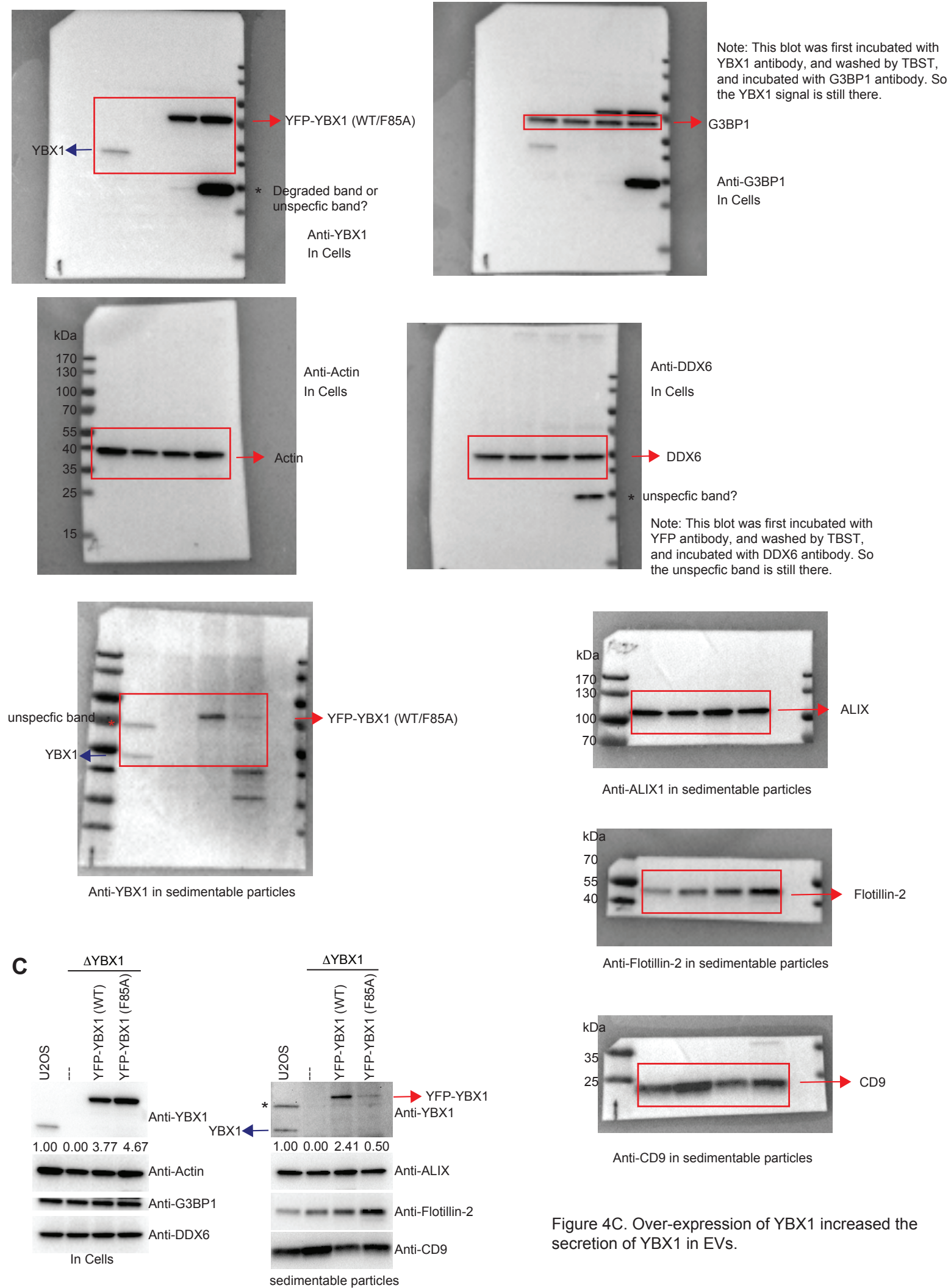

Figure 4C. Over-expression of YBX1 increased the secretion of YBX1 in EVs.

Supplement: Figure 4—source data 1. [file elife-71982-fig4-data1.zip › Figure 4-source data 1 for Figure 4C/Uncropped Western blot images corresponding to Figure 4C.pdf]

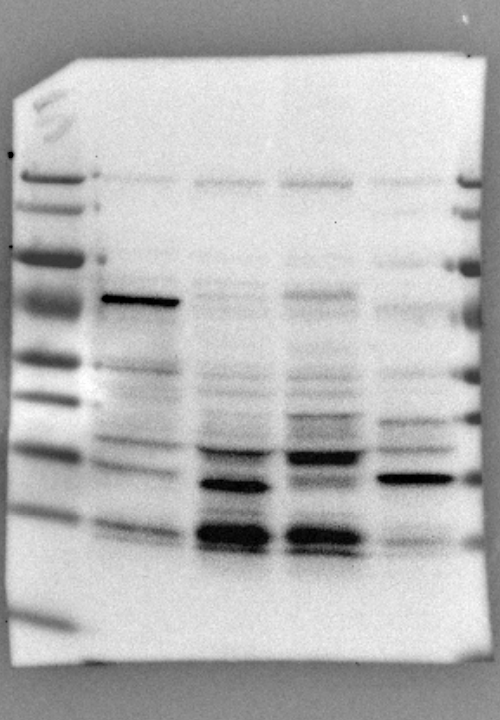

Supplement: Figure 4—source data 2. [file elife-71982-fig4-data2.zip › Figure 4-source data 2 for Figure 4D/Figure 4D-raw unedited blot for YFP in sedimentable particles merged with marker.tif]

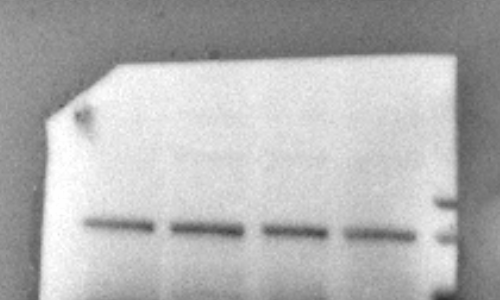

Supplement: Figure 4—source data 2. [file elife-71982-fig4-data2.zip › Figure 4-source data 2 for Figure 4D/Figure 4D-raw unedited blot for GM130 in cells merged with marker.tif]

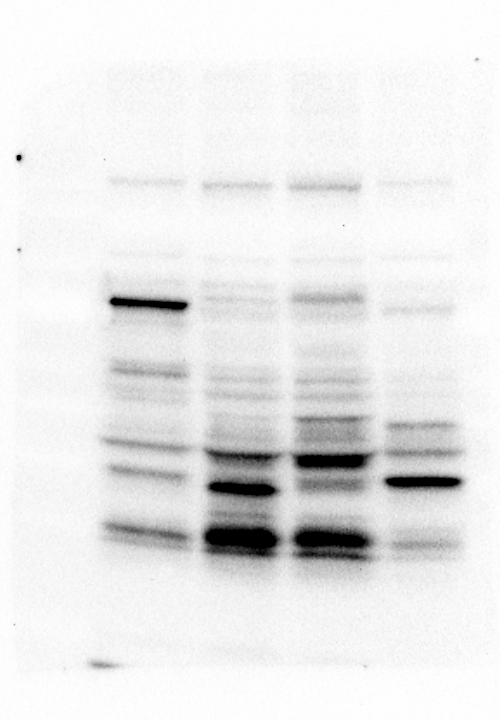

Supplement: Figure 4—source data 2. [file elife-71982-fig4-data2.zip › Figure 4-source data 2 for Figure 4D/Figure 4D-raw unedited blot for YFP in sedimentable particles without marker.tif]

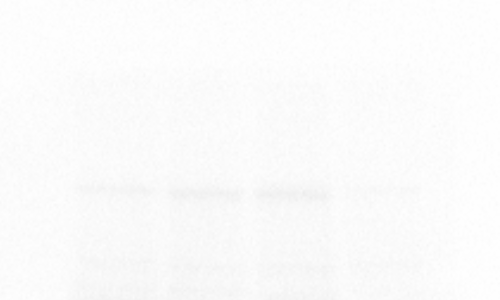

Supplement: Figure 4—source data 2. [file elife-71982-fig4-data2.zip › Figure 4-source data 2 for Figure 4D/Figure 4D-raw unedited blot for GM130 in sedimentable particles without marker.tif]

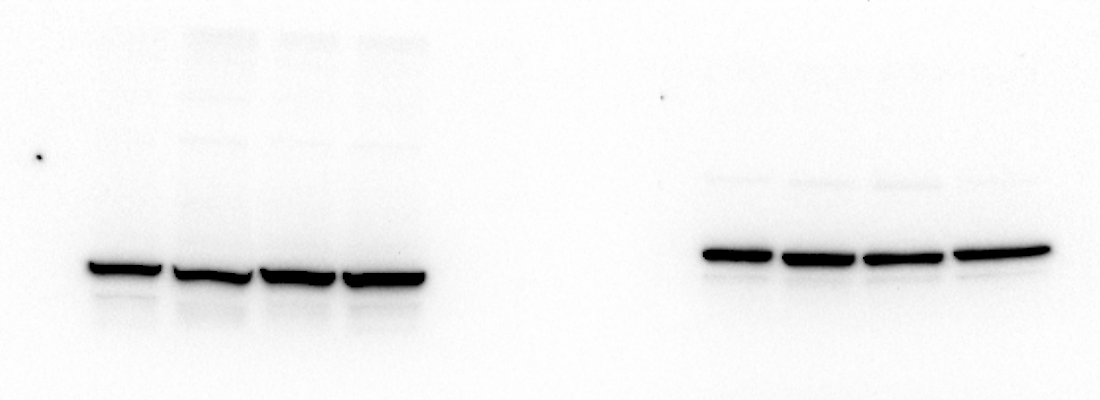

Supplement: Figure 4—source data 2. [file elife-71982-fig4-data2.zip › Figure 4-source data 2 for Figure 4D/Figure 4D-raw unedited blot for ALIX in cells and sedimentable particles without marker.tif]

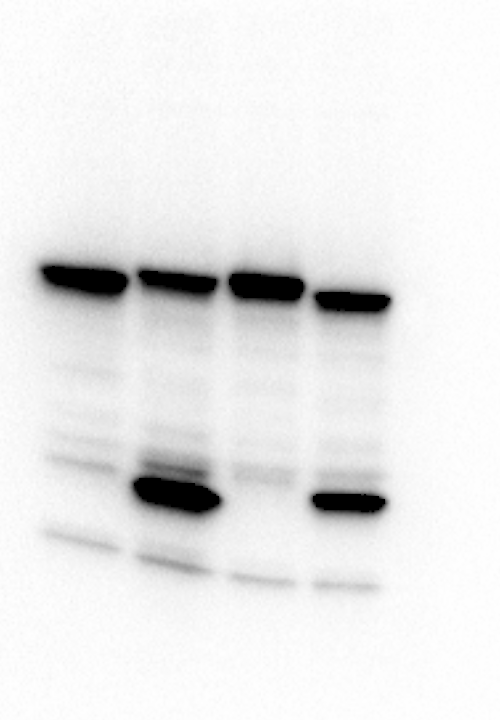

Supplement: Figure 4—source data 2. [file elife-71982-fig4-data2.zip › Figure 4-source data 2 for Figure 4D/Figure 4D-raw unedited blot for YFP in cells without marker.tif]

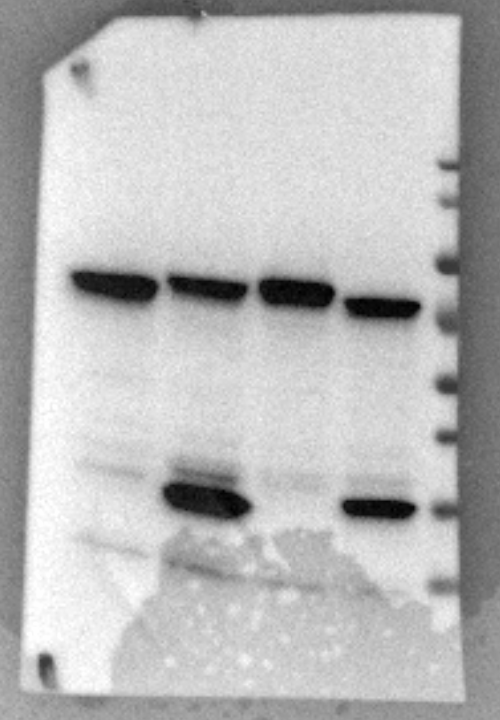

Supplement: Figure 4—source data 2. [file elife-71982-fig4-data2.zip › Figure 4-source data 2 for Figure 4D/Figure 4D-raw unedited blot for YFP in cells merged with marker.tif]

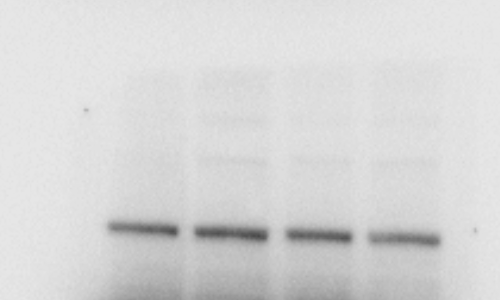

Supplement: Figure 4—source data 2. [file elife-71982-fig4-data2.zip › Figure 4-source data 2 for Figure 4D/Figure 4D-raw unedited blot for GM130 in cells without marker.tif]

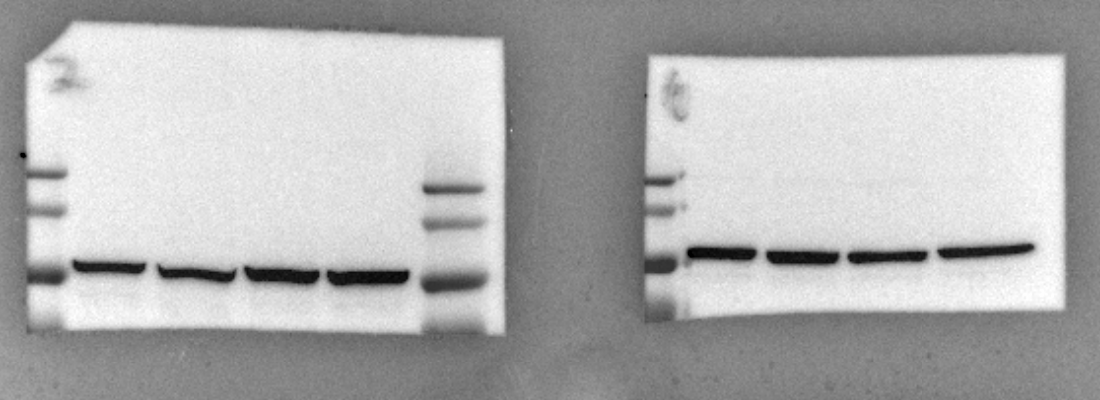

Supplement: Figure 4—source data 2. [file elife-71982-fig4-data2.zip › Figure 4-source data 2 for Figure 4D/Figure 4D-raw unedited blot for ALIX in cells and sedimentable particles merged with marker.tif]

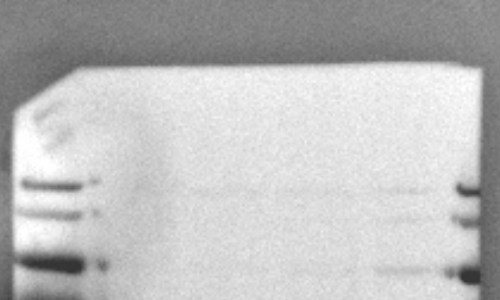

Supplement: Figure 4—source data 2. [file elife-71982-fig4-data2.zip › Figure 4-source data 2 for Figure 4D/Figure 4D-raw unedited blot for GM130 in sedimentable particles merged with marker.tif]

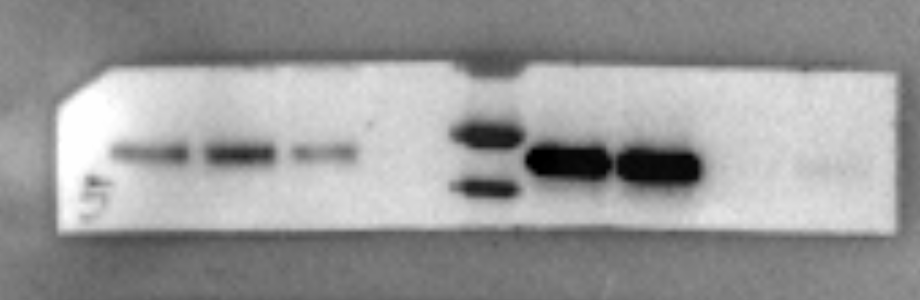

Supplement: Figure 4—source data 3. [file elife-71982-fig4-data3.zip › Figure 4-source data 3 for figure 4E/Figure 4E-raw unedited blot for Flotillin-2 merged with protein marker.tif]

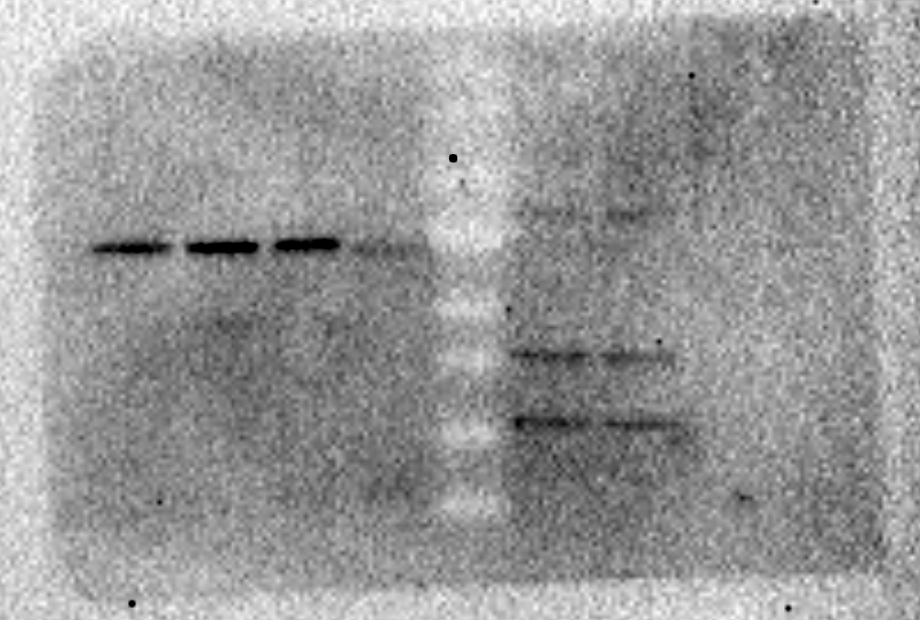

Supplement: Figure 4—source data 3. [file elife-71982-fig4-data3.zip › Figure 4-source data 3 for figure 4E/Figure 4E-raw unedited blot for YBX1 without protein marker.tif]

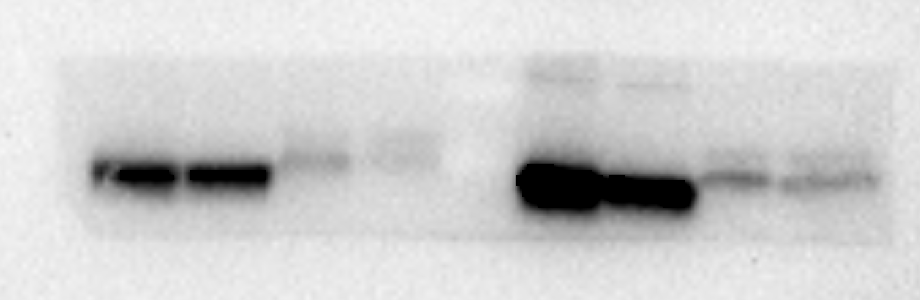

Supplement: Figure 4—source data 3. [file elife-71982-fig4-data3.zip › Figure 4-source data 3 for figure 4E/Figure 4E-raw unedited blot for CD9 without protein marker.tif]

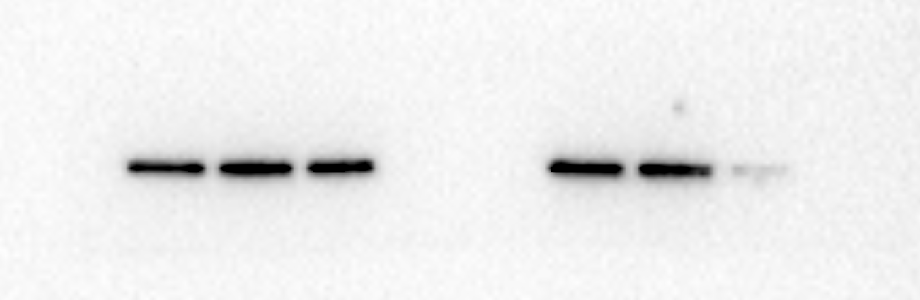

Supplement: Figure 4—source data 3. [file elife-71982-fig4-data3.zip › Figure 4-source data 3 for figure 4E/Figure 4E-raw unedited blot for ALIX without protein marker.tif]

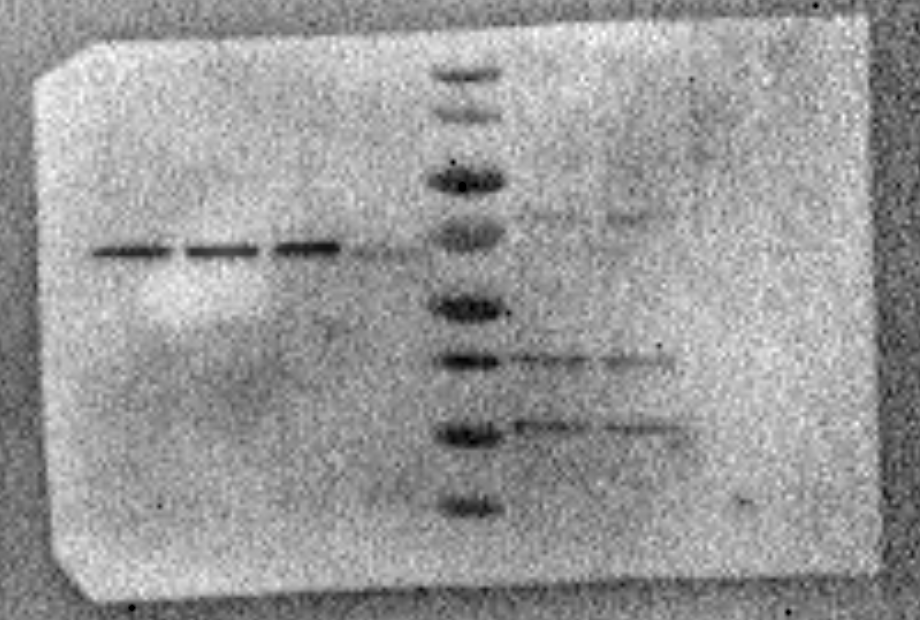

Supplement: Figure 4—source data 3. [file elife-71982-fig4-data3.zip › Figure 4-source data 3 for figure 4E/Figure 4E-raw unedited blot for YBX1 merged with protein marker.tif]

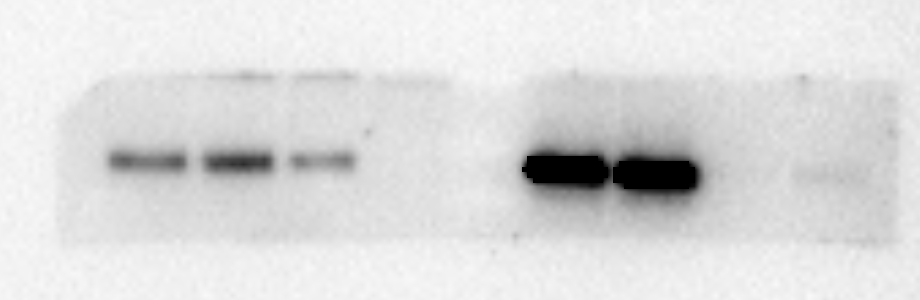

Supplement: Figure 4—source data 3. [file elife-71982-fig4-data3.zip › Figure 4-source data 3 for figure 4E/Figure 4E-raw unedited blot for Flotillin-2 without protein marker.tif]

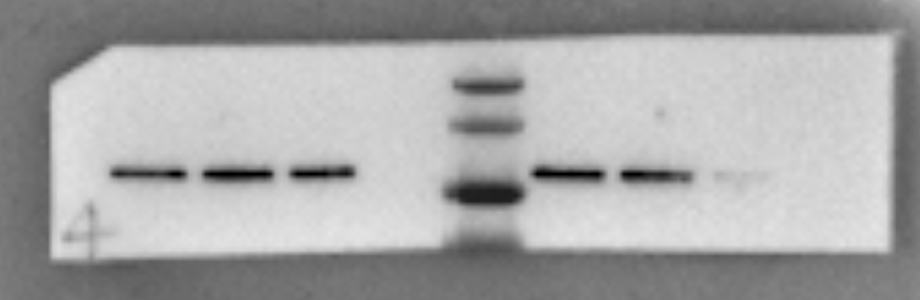

Supplement: Figure 4—source data 3. [file elife-71982-fig4-data3.zip › Figure 4-source data 3 for figure 4E/Figure 4E-raw unedited blot for ALIX merged with protein marker.tif]

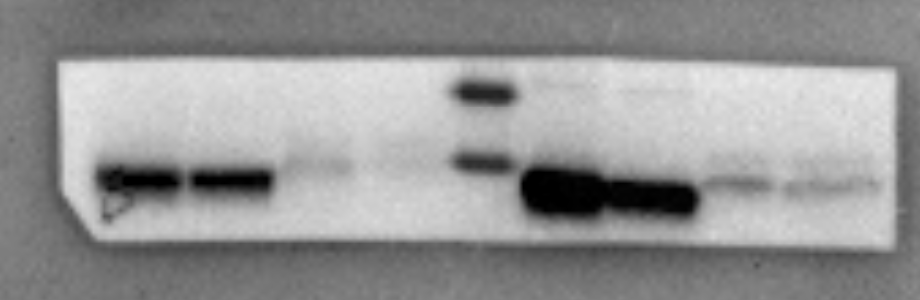

Supplement: Figure 4—source data 3. [file elife-71982-fig4-data3.zip › Figure 4-source data 3 for figure 4E/Figure 4E-raw unedited blot for CD9 merged with protein marker.tif]

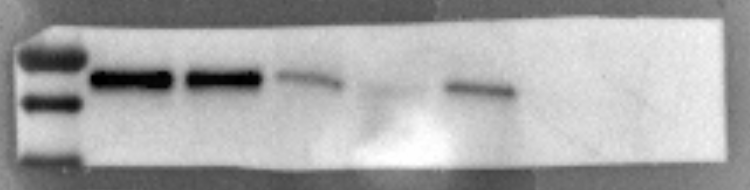

Supplement: Figure 4—source data 4. [file elife-71982-fig4-data4.zip › Figure 4-source data 4 for Figure 4F/Figure 4F-raw unedited blot for Flotillin-2 merged with protein marker.tif]

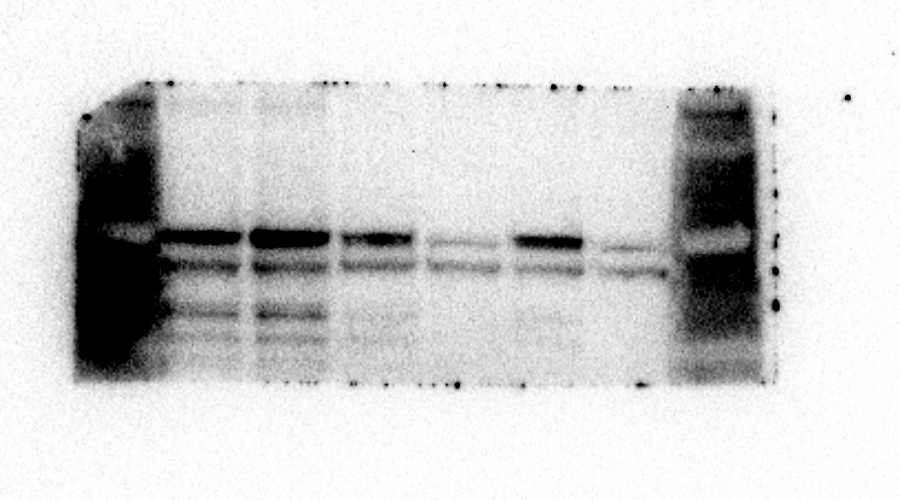

Supplement: Figure 4—source data 4. [file elife-71982-fig4-data4.zip › Figure 4-source data 4 for Figure 4F/Figure 4F-raw unedited blot for YBX1.tif]

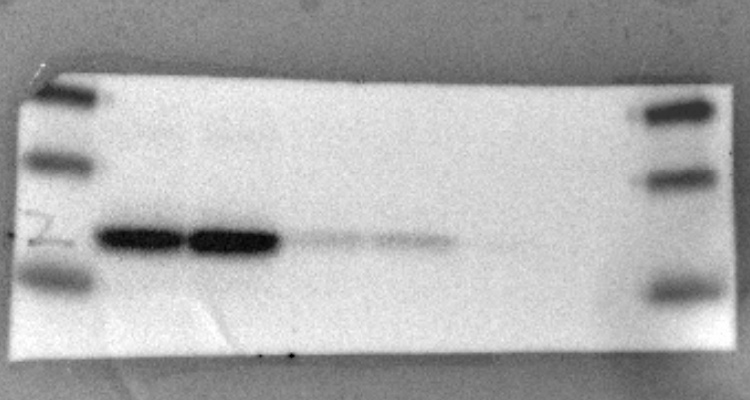

Supplement: Figure 4—source data 4. [file elife-71982-fig4-data4.zip › Figure 4-source data 4 for Figure 4F/Figure 4F-raw unedited blot for CD9 merged with protein marker.tif]

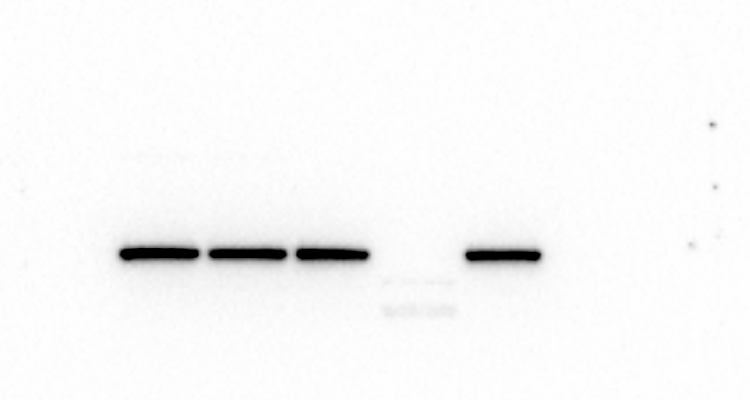

Supplement: Figure 4—source data 4. [file elife-71982-fig4-data4.zip › Figure 4-source data 4 for Figure 4F/Figure 4F-raw unedited blot for ALIX without protein marker.tif]

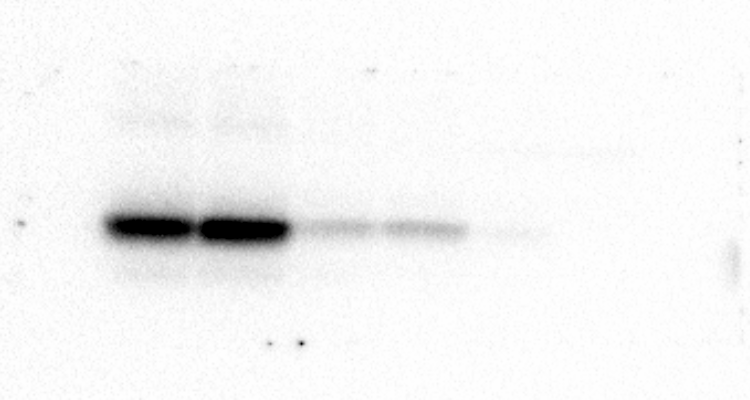

Supplement: Figure 4—source data 4. [file elife-71982-fig4-data4.zip › Figure 4-source data 4 for Figure 4F/Figure 4F-raw unedited blot for CD9 without protein marker.tif]

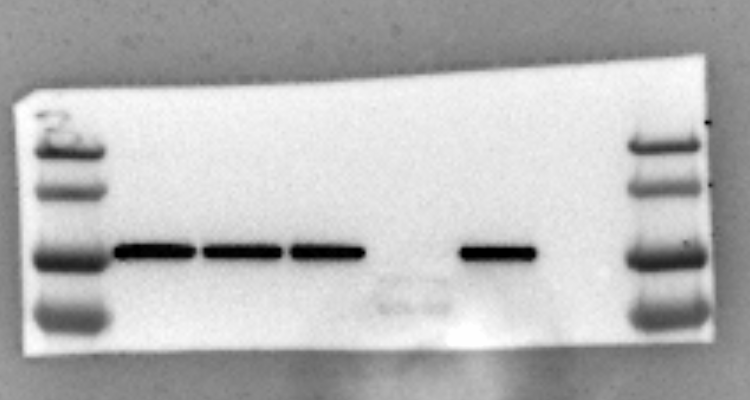

Supplement: Figure 4—source data 4. [file elife-71982-fig4-data4.zip › Figure 4-source data 4 for Figure 4F/Figure 4F-raw unedited blot for ALIX merged with protein marker.tif]

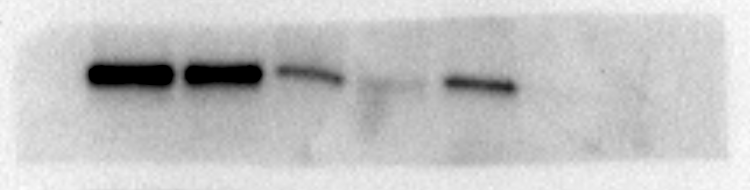

Supplement: Figure 4—source data 4. [file elife-71982-fig4-data4.zip › Figure 4-source data 4 for Figure 4F/Figure 4F-raw unedited blot for Flotillin-2 without protein marker.tif]

Figure 4I

uncropped blots

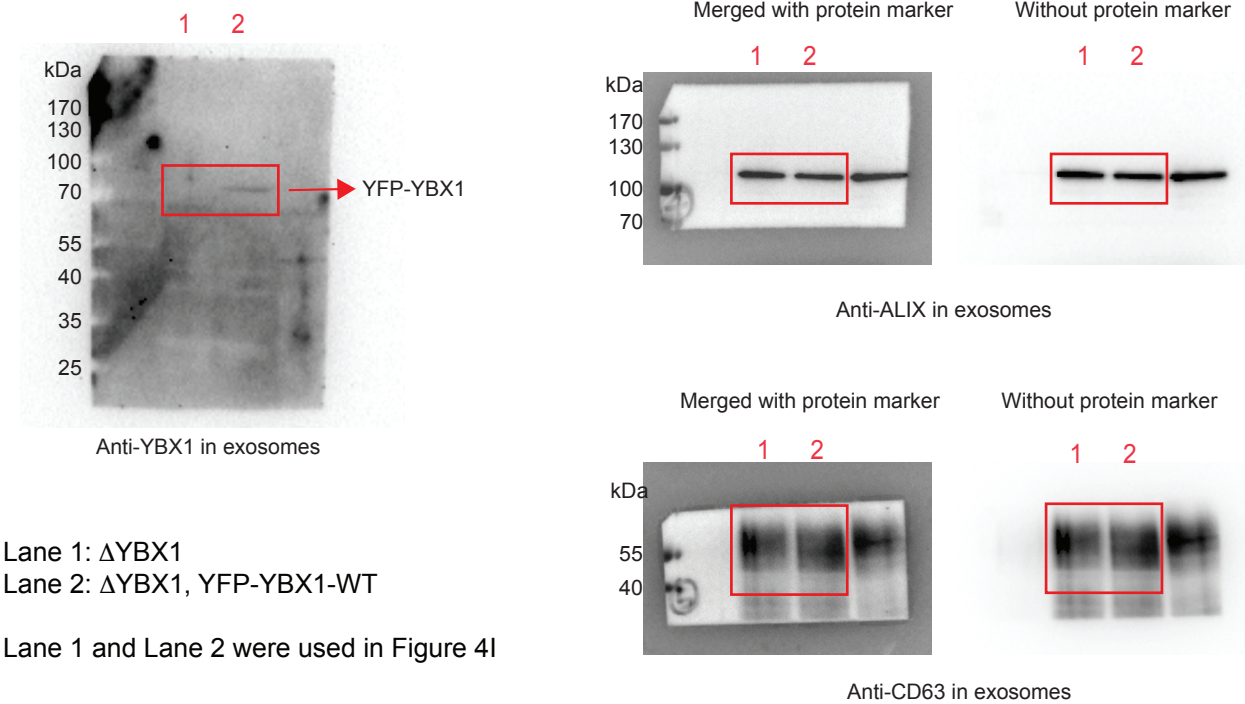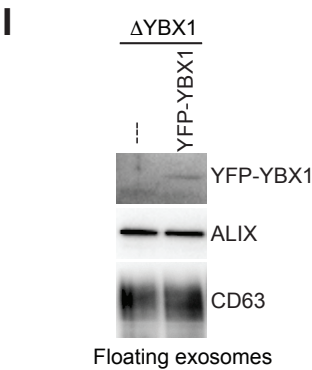

Figure 4I. YFP-YBX1 detected in sucrose post-flotation fraction.

Supplement: Figure 4—source data 5. [file elife-71982-fig4-data5.zip › Figure 4-source data 5 for Figure 4I/Uncropped Western blot images corresponding to Figure 4I.pdf]

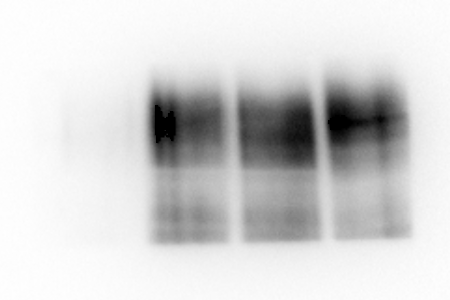

Supplement: Figure 4—source data 5. [file elife-71982-fig4-data5.zip › Figure 4-source data 5 for Figure 4I/Figure 4I-raw unedited blot for CD63 without protein marker.tif]

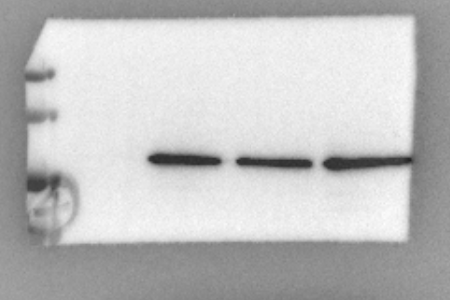

Supplement: Figure 4—source data 5. [file elife-71982-fig4-data5.zip › Figure 4-source data 5 for Figure 4I/Figure 4I-raw unedited blot for ALIX merged with protein marker.tif]

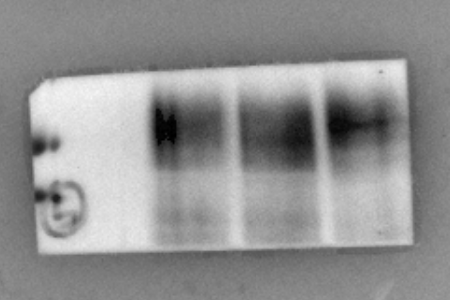

Supplement: Figure 4—source data 5. [file elife-71982-fig4-data5.zip › Figure 4-source data 5 for Figure 4I/Figure 4I-raw unedited blot for CD63 merged with protein marker.tif]

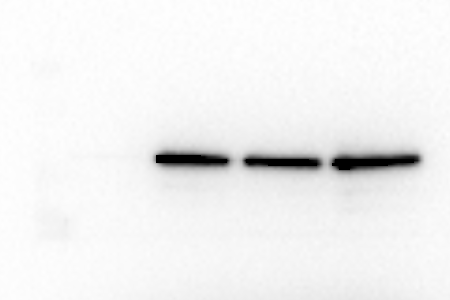

Supplement: Figure 4—source data 5. [file elife-71982-fig4-data5.zip › Figure 4-source data 5 for Figure 4I/Figure 4I-raw unedited blot for ALIX without protein marker.tif]

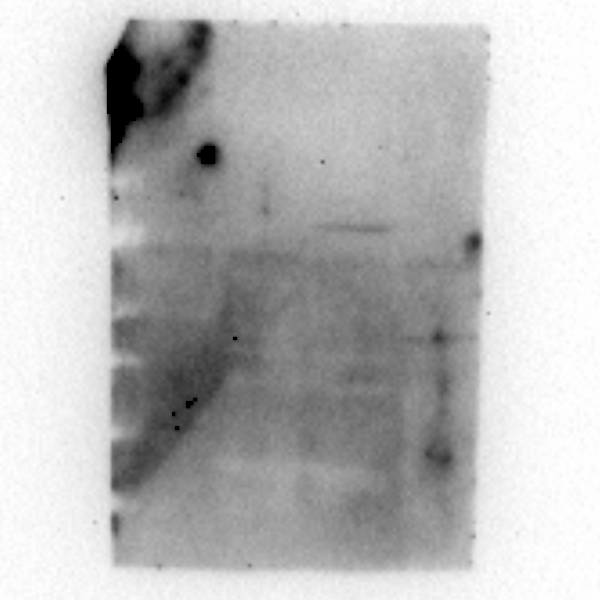

Supplement: Figure 4—source data 5. [file elife-71982-fig4-data5.zip › Figure 4-source data 5 for Figure 4I/Figure 4I-raw unedited blot for YBX1.tif]

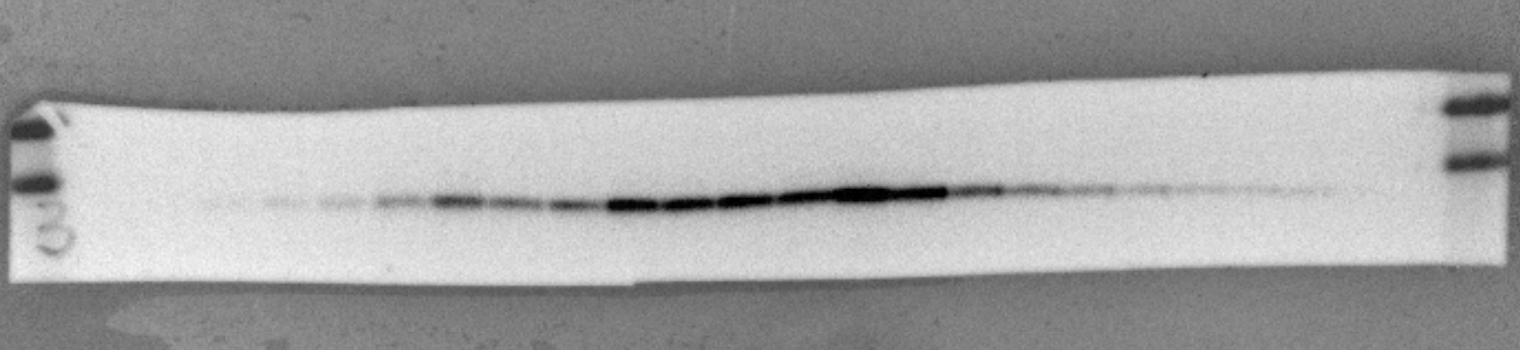

Supplement: Figure 4—source data 6. [file elife-71982-fig4-data6.zip › Figure 4-source data 6 for Figure 4K/Figure 4K-raw unedited blot for CD9 merged with protein marker.tif]

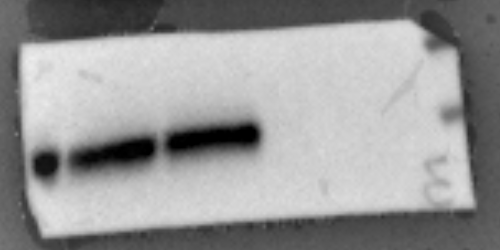

Supplement: Figure 4—source data 6. [file elife-71982-fig4-data6.zip › Figure 4-source data 6 for Figure 4K/Figure 4K-raw unedited blot for combined CD9 merged with protein marker.tif]

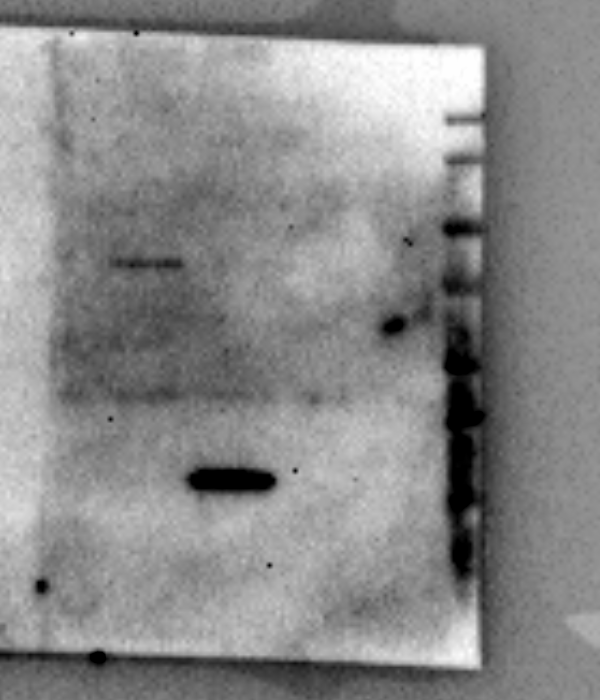

Supplement: Figure 4—source data 6. [file elife-71982-fig4-data6.zip › Figure 4-source data 6 for Figure 4K/Figure 4K-raw unedited blot for YBX1 merged with protein marker.tif]

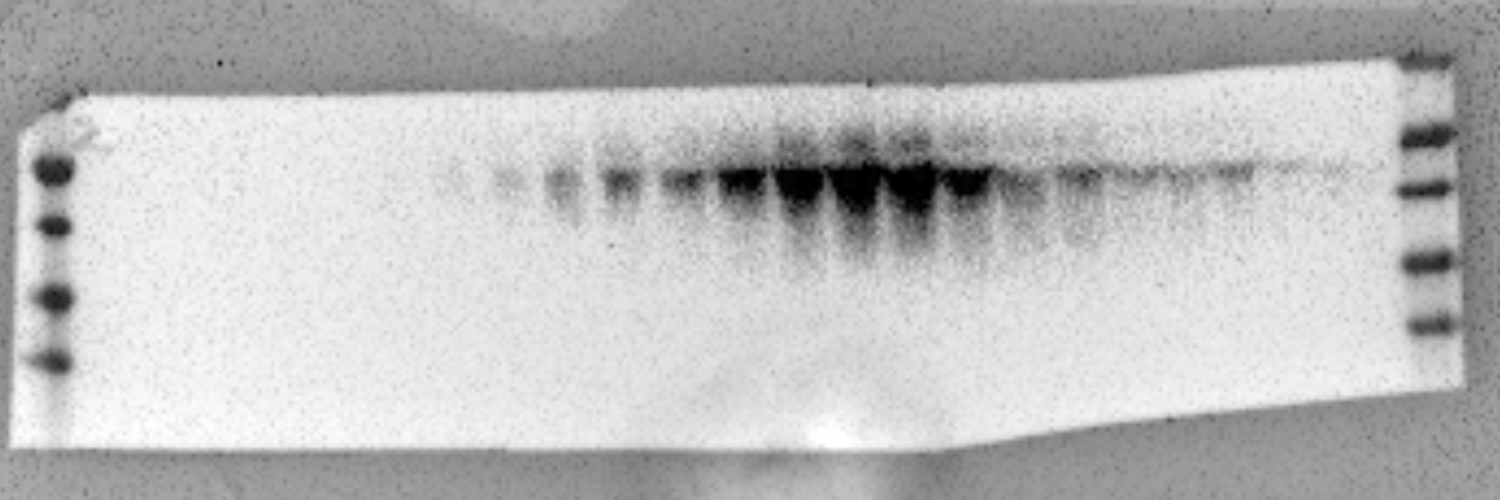

Supplement: Figure 4—source data 6. [file elife-71982-fig4-data6.zip › Figure 4-source data 6 for Figure 4K/Figure 4K-raw unedited blot for CD63 merged with protein marker.tif]

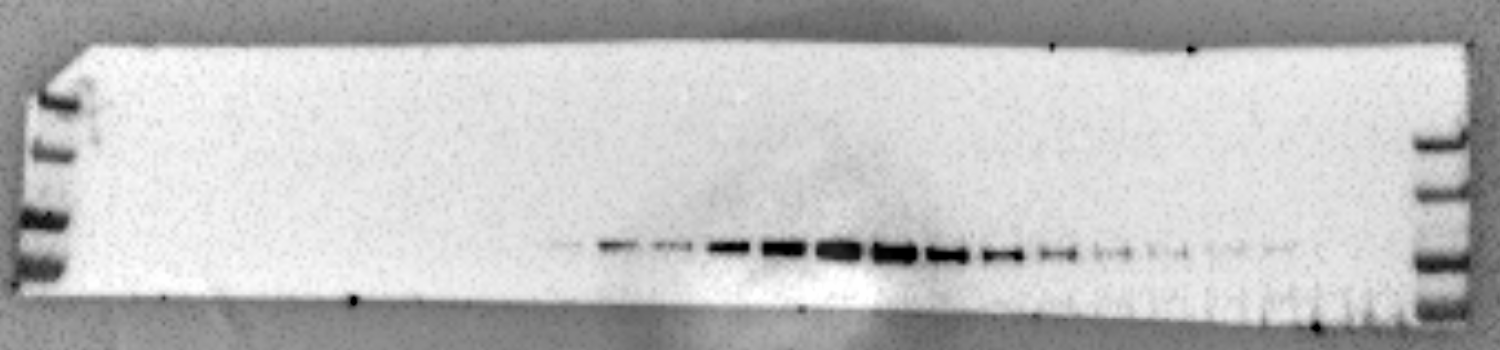

Supplement: Figure 4—source data 6. [file elife-71982-fig4-data6.zip › Figure 4-source data 6 for Figure 4K/Figure 4K-raw unedited blot for ALIX merged with protein marker.tif]

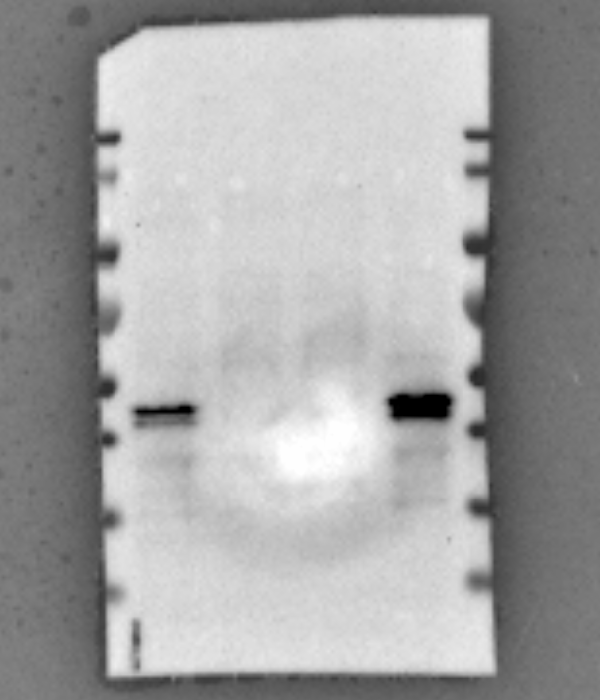

Supplement: Figure 5—source data 1. [file elife-71982-fig5-data1.zip › Figure 5-source data 1 for Figure 5C/Figure 5C-raw unedited blot for YBX1 merged with protein marker.tif]

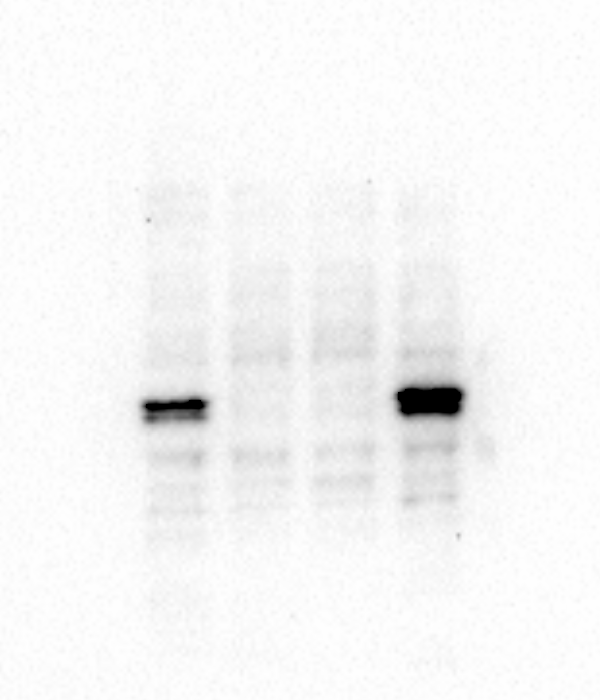

Supplement: Figure 5—source data 1. [file elife-71982-fig5-data1.zip › Figure 5-source data 1 for Figure 5C/Figure 5C-raw unedited blot for YBX1 without protein marker.tif]

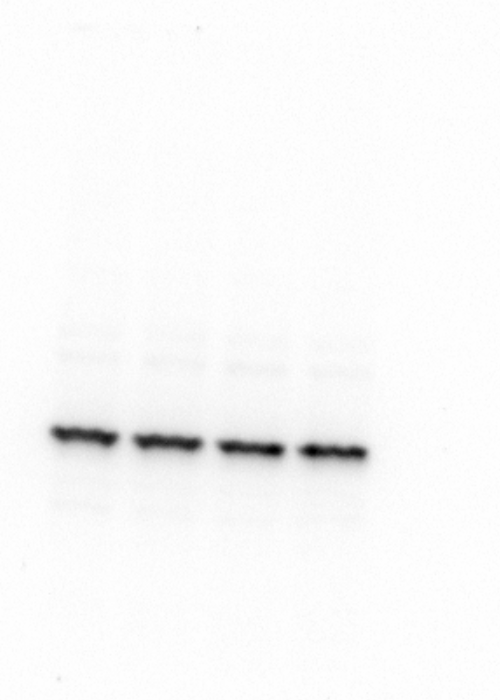

Supplement: Figure 5—source data 1. [file elife-71982-fig5-data1.zip › Figure 5-source data 1 for Figure 5C/Figure 5C-raw unedited blot for Actin without protein marker.tif]

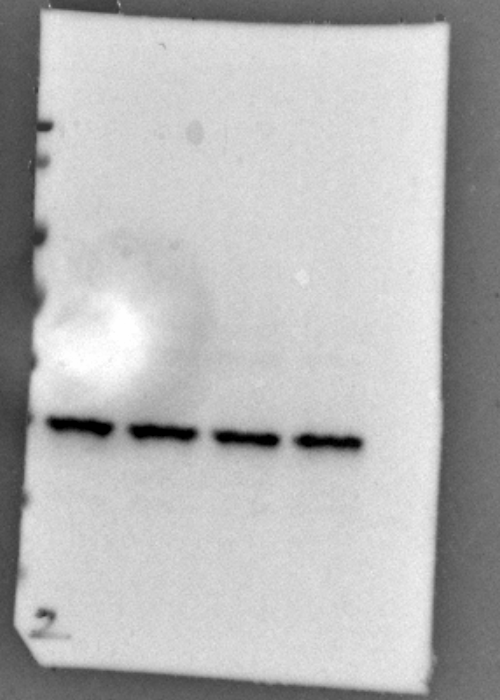

Supplement: Figure 5—source data 1. [file elife-71982-fig5-data1.zip › Figure 5-source data 1 for Figure 5C/Figure 5C-raw unedited blot for Actin merged with protein marker.tif]

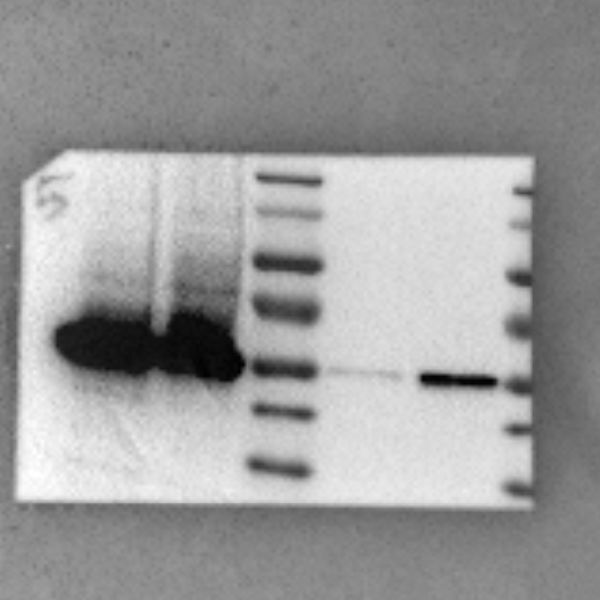

Supplement: Figure 7—source data 3. [file elife-71982-fig7-data3.zip › Figure 7-source data 3 for Figure 7G/Figure 7G-unmodified blot for DDX6-IP-merged with protein marker.tif]

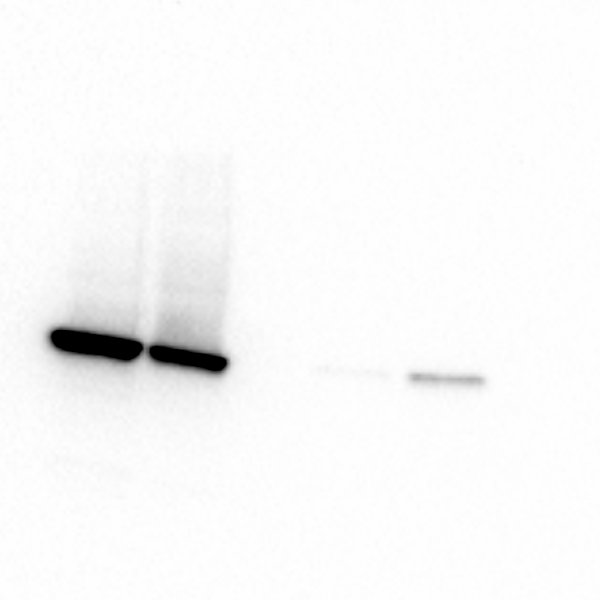

Supplement: Figure 7—source data 3. [file elife-71982-fig7-data3.zip › Figure 7-source data 3 for Figure 7G/Figure 7G-unmodified blot for DDX6-Input without protein marker.tif]

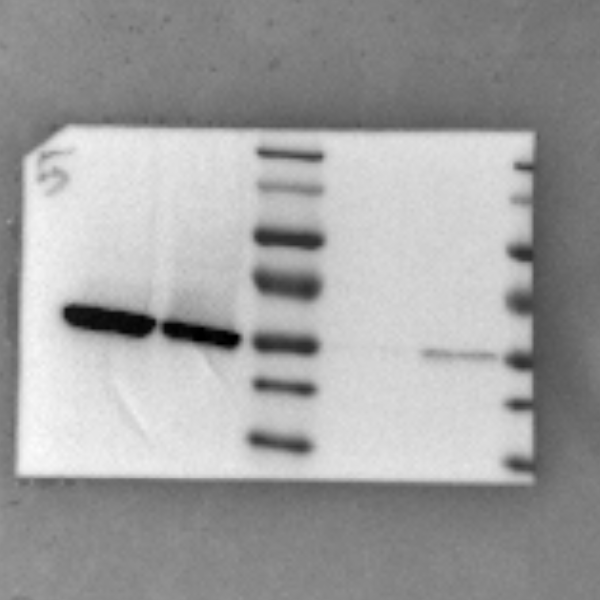

Supplement: Figure 7—source data 3. [file elife-71982-fig7-data3.zip › Figure 7-source data 3 for Figure 7G/Figure 7G-unmodified blot for DDX6-Input merged with protein marker.tif]

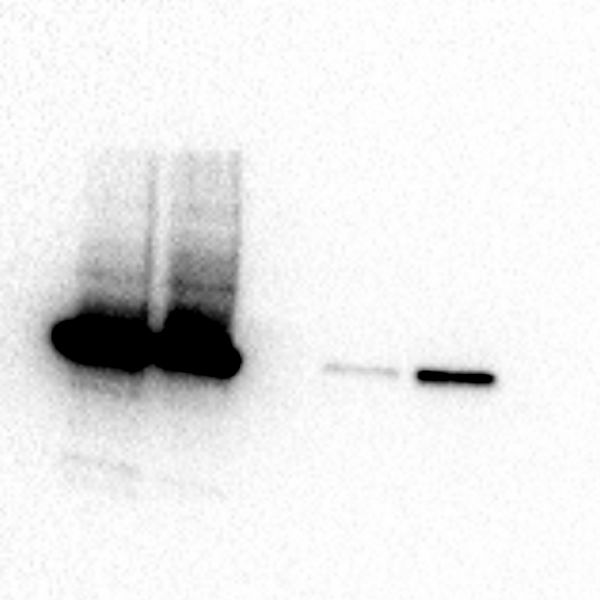

Supplement: Figure 7—source data 3. [file elife-71982-fig7-data3.zip › Figure 7-source data 3 for Figure 7G/Figure 7G-unmodified blot for DDX6-IP-without protein marker.tif]

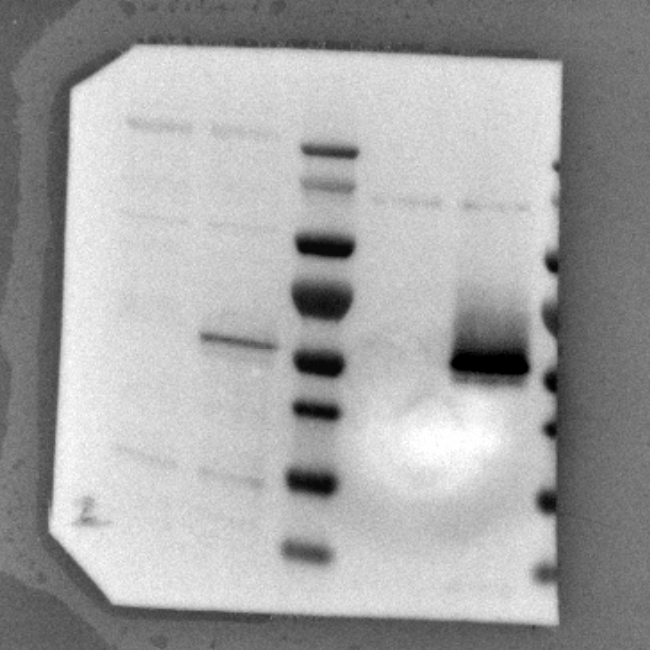

Supplement: Figure 7—source data 3. [file elife-71982-fig7-data3.zip › Figure 7-source data 3 for Figure 7G/Figure 7G-unmodified blot for Flag merged with protein marker.tif]

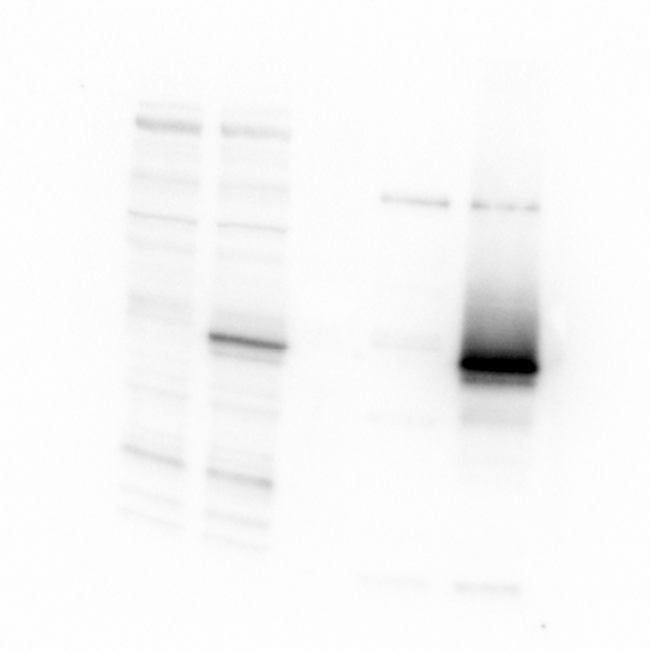

Supplement: Figure 7—source data 3. [file elife-71982-fig7-data3.zip › Figure 7-source data 3 for Figure 7G/Figure 7G-unmodified blot for Flag without protein marker.tif]

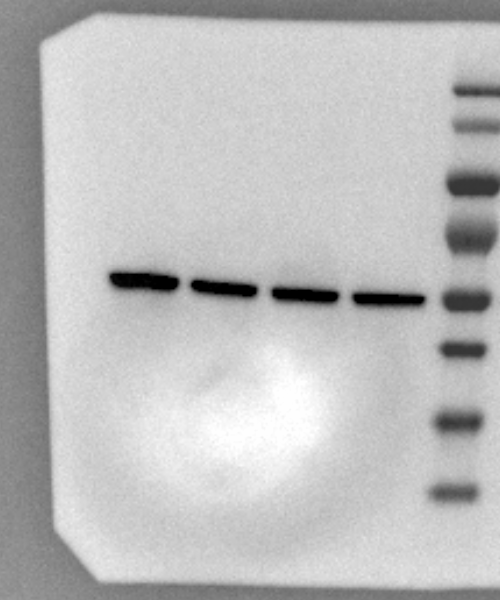

Supplement: Figure 7—source data 4. [file elife-71982-fig7-data4.zip › Figure 7-source data 4 for Figure 7H/Figure 7H-unmodified blot for DDX6-Input merged with protein marker.tif]

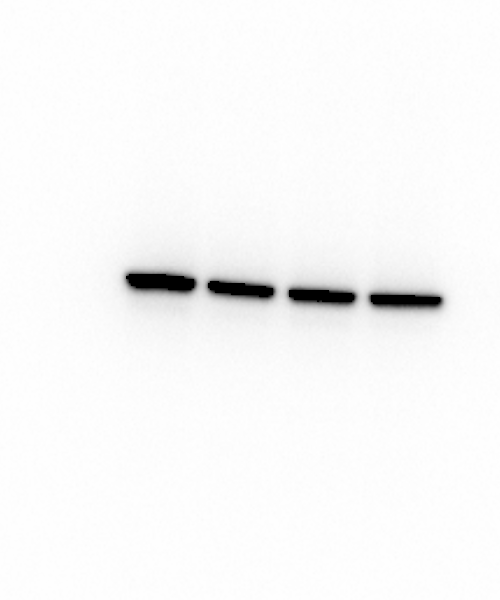

Supplement: Figure 7—source data 4. [file elife-71982-fig7-data4.zip › Figure 7-source data 4 for Figure 7H/Figure 7H-unmodified blot for DDX6-Input without protein marker.tif]

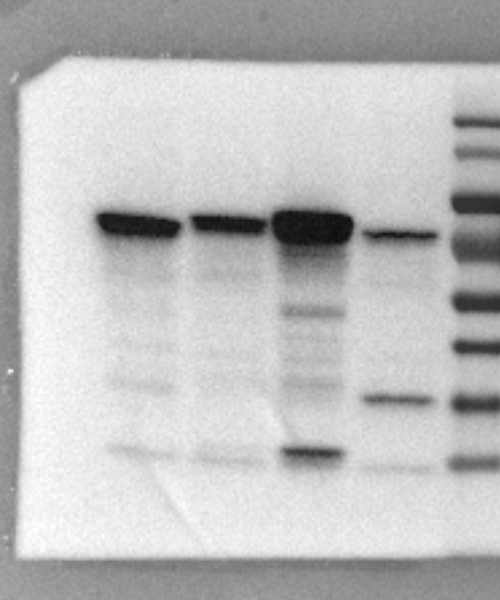

Supplement: Figure 7—source data 4. [file elife-71982-fig7-data4.zip › Figure 7-source data 4 for Figure 7H/Figure 7H-unmodified blot for YFP-Input merged with protein marker.tif]

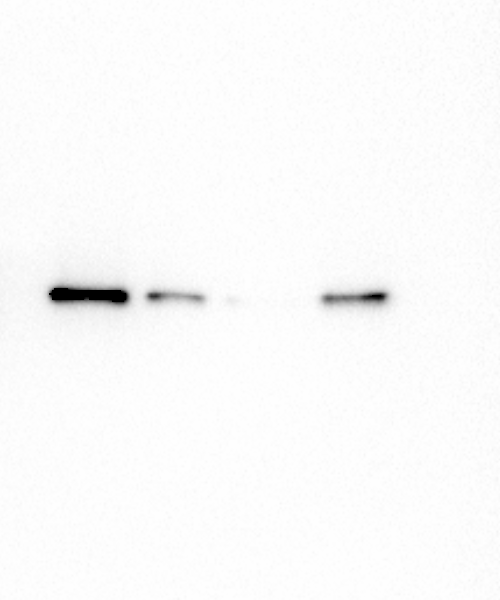

Supplement: Figure 7—source data 4. [file elife-71982-fig7-data4.zip › Figure 7-source data 4 for Figure 7H/Figure 7H-unmodified blot for DDX6-IP without protein marker.tif]

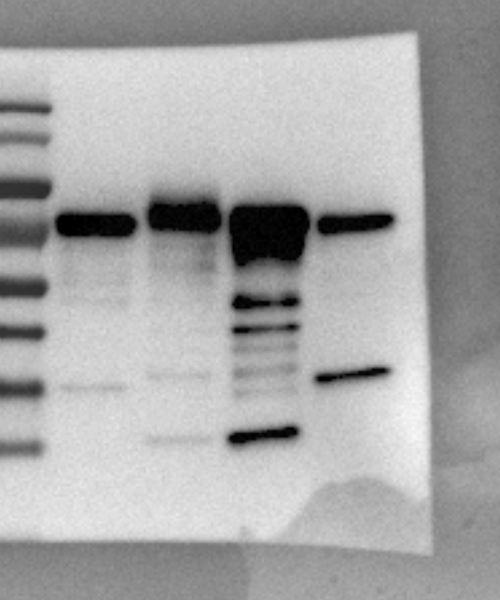

Supplement: Figure 7—source data 4. [file elife-71982-fig7-data4.zip › Figure 7-source data 4 for Figure 7H/Figure 7H-unmodified blot for YFP-IP merged with protein marker.tif]

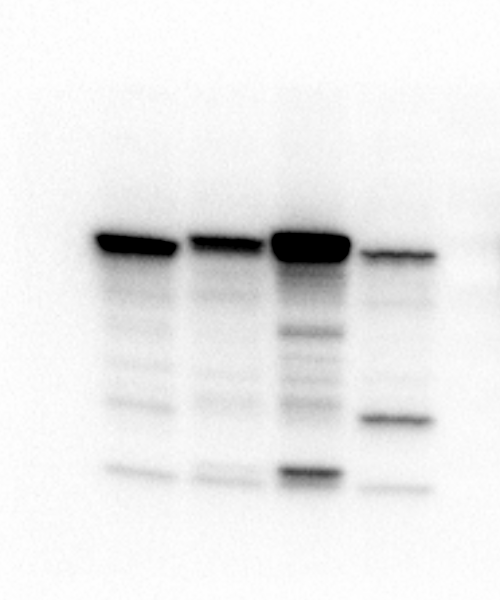

Supplement: Figure 7—source data 4. [file elife-71982-fig7-data4.zip › Figure 7-source data 4 for Figure 7H/Figure 7H-unmodified blot for YFP-Input without protein marker.tif]

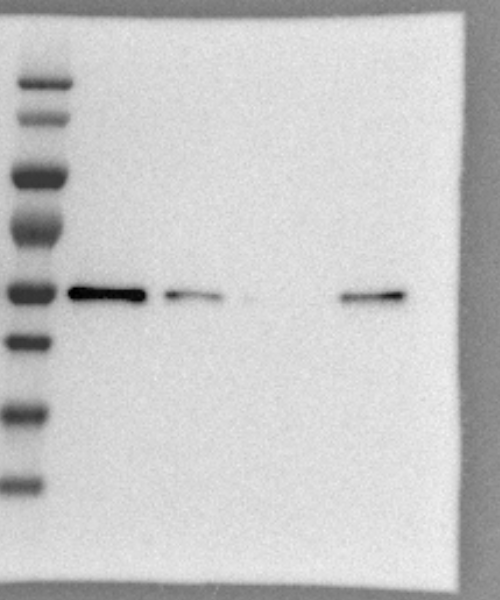

Supplement: Figure 7—source data 4. [file elife-71982-fig7-data4.zip › Figure 7-source data 4 for Figure 7H/Figure 7H-unmodified blot for DDX6-IP merged with protein marker.tif]

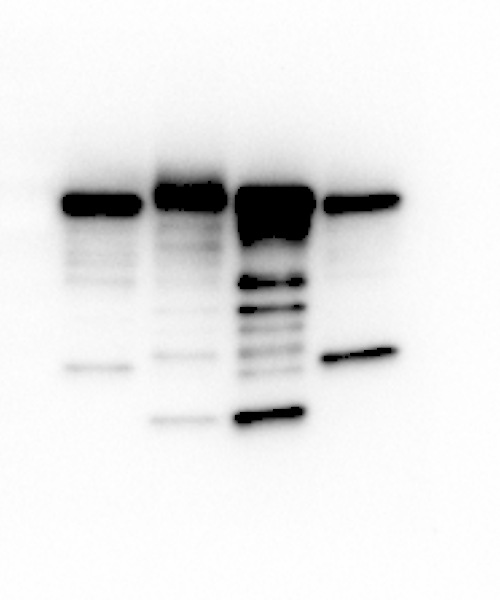

Supplement: Figure 7—source data 4. [file elife-71982-fig7-data4.zip › Figure 7-source data 4 for Figure 7H/Figure 7H-unmodified blot for YFP-IP without protein marker.tif]

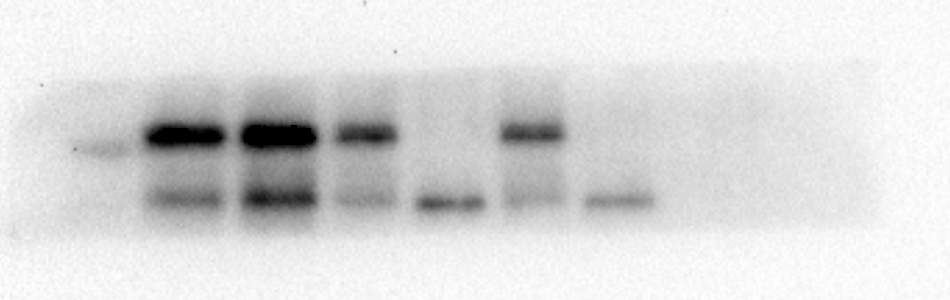

Supplement: Figure 7—source data 5. [file elife-71982-fig7-data5.zip › Figure 7-source data 5 for Figure 7I/Figure 7I-unmodified blot for DDX6-without protein marker.tif]

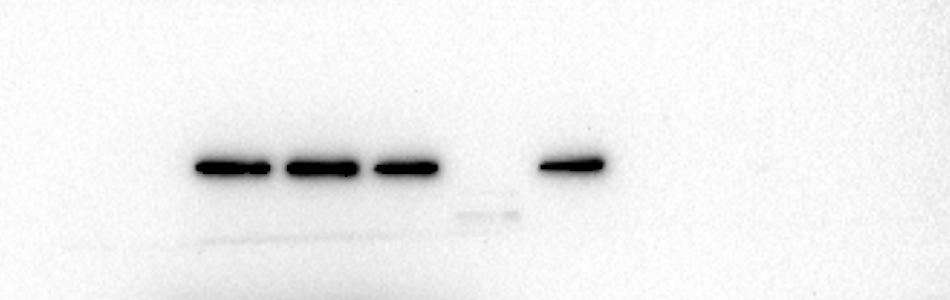

Supplement: Figure 7—source data 5. [file elife-71982-fig7-data5.zip › Figure 7-source data 5 for Figure 7I/Figure 7I-unmodified blot for ALIX-without protein marker.tif]

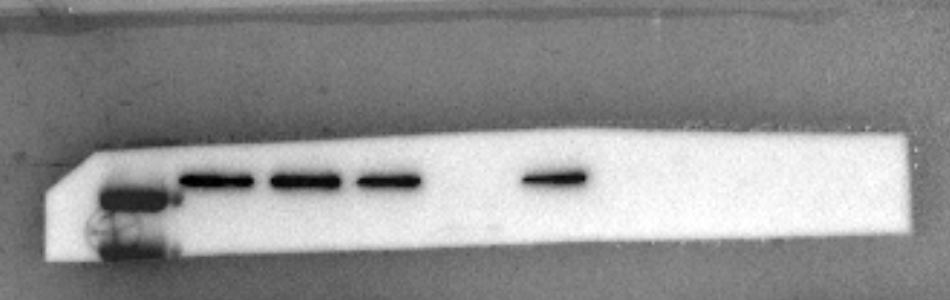

Supplement: Figure 7—source data 5. [file elife-71982-fig7-data5.zip › Figure 7-source data 5 for Figure 7I/Figure 7I-unmodified blot for ALIX-merged with protein marker.tif]

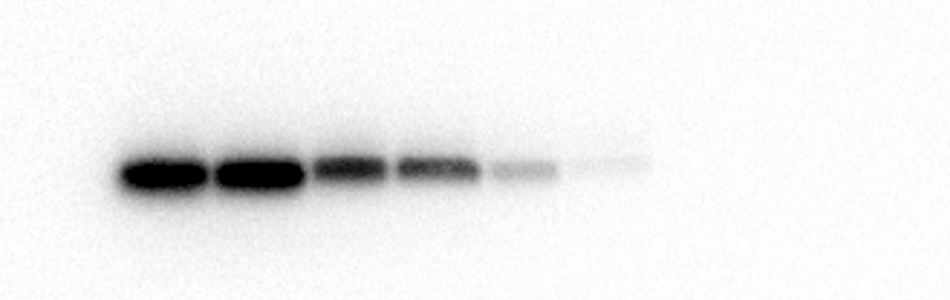

Supplement: Figure 7—source data 5. [file elife-71982-fig7-data5.zip › Figure 7-source data 5 for Figure 7I/Figure 7I-unmodified blot for CD9-without protein marker.tif]

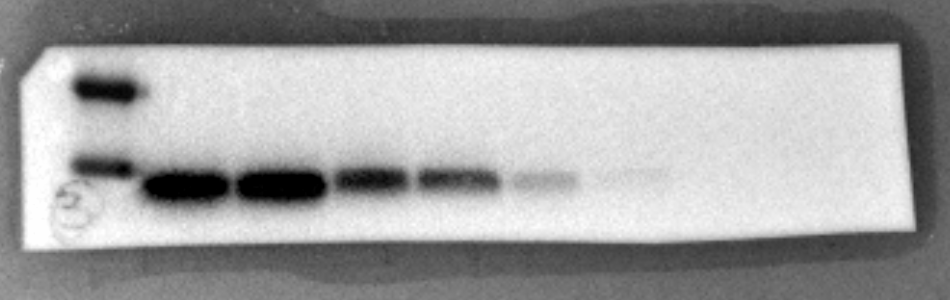

Supplement: Figure 7—source data 5. [file elife-71982-fig7-data5.zip › Figure 7-source data 5 for Figure 7I/Figure 7I-unmodified blot for CD9-merged with protein marker.tif]

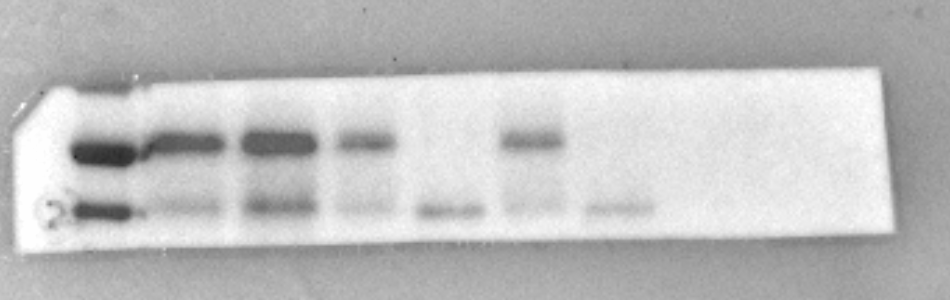

Supplement: Figure 7—source data 5. [file elife-71982-fig7-data5.zip › Figure 7-source data 5 for Figure 7I/Figure 7I-unmodified blot for DDX6-merged with protein marker.tif]

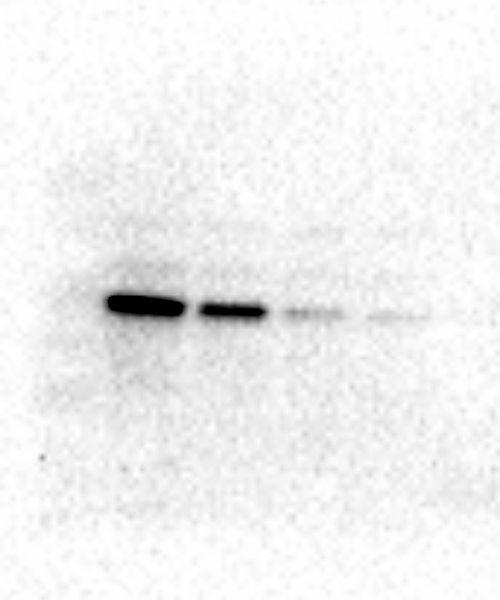

Supplement: Figure 7—figure supplement 3—source data 1. [file elife-71982-fig7-figsupp3-data1.zip › Figure 7-supplement 3-source data 1/unedited blot for LSM14A.tif]

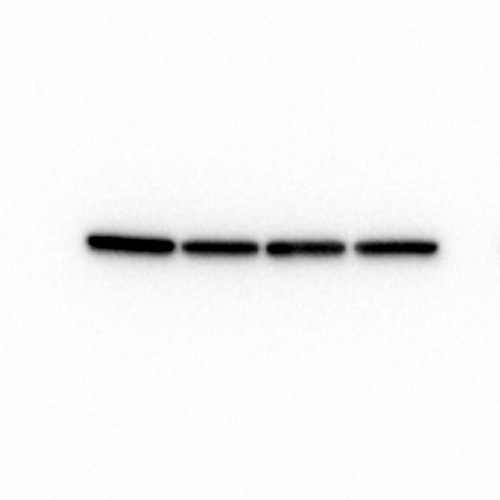

Supplement: Figure 7—figure supplement 3—source data 1. [file elife-71982-fig7-figsupp3-data1.zip › Figure 7-supplement 3-source data 1/unedited blot for actin related to 4E-T.tif]

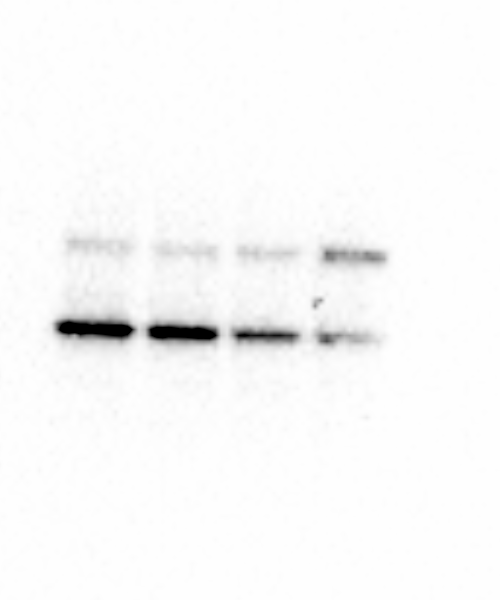

Supplement: Figure 7—figure supplement 3—source data 1. [file elife-71982-fig7-figsupp3-data1.zip › Figure 7-supplement 3-source data 1/unedited blot for DDX6.tif]

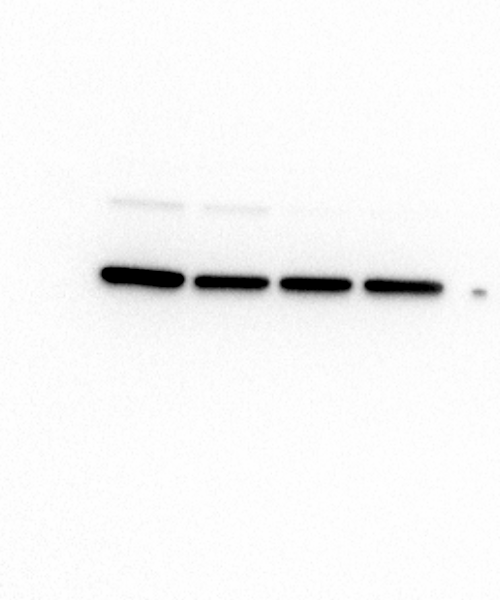

Supplement: Figure 7—figure supplement 3—source data 1. [file elife-71982-fig7-figsupp3-data1.zip › Figure 7-supplement 3-source data 1/unedited blot for Actin related to LSM14A.tif]

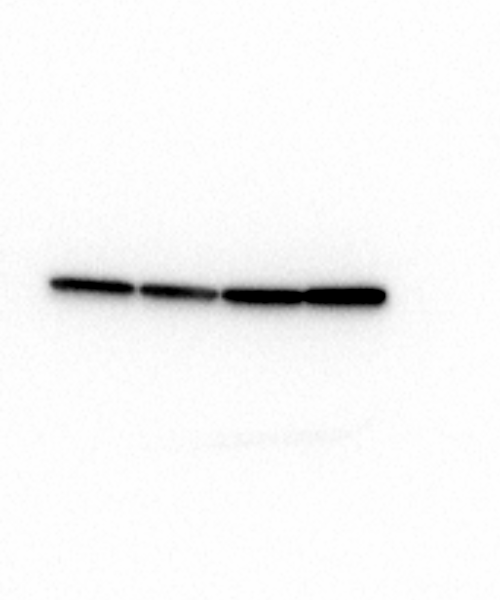

Supplement: Figure 7—figure supplement 3—source data 1. [file elife-71982-fig7-figsupp3-data1.zip › Figure 7-supplement 3-source data 1/unedited blot for Actin related to DDX6.tif]

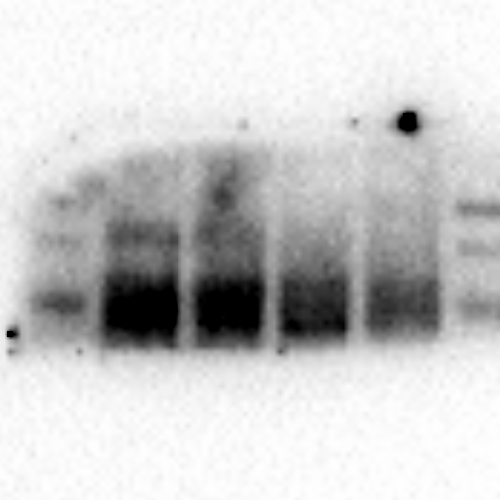

Supplement: Figure 7—figure supplement 3—source data 1. [file elife-71982-fig7-figsupp3-data1.zip › Figure 7-supplement 3-source data 1/unedited blot for 4E-T.tif]
