# Supplementary material for: Selective sorting of microRNAs into exosomes by phase-separated YBX1 condensates
Source: eLife. 2021 Nov 12;10:e71982. doi: 10.7554/eLife.71982 (PMC8612733; doi:10.7554/eLife.71982)
Supplement: Figure 4—source data 2. [file elife-71982-fig4-data2.zip › Figure 4-source data 2 for Figure 4D/Uncropped Western blot images corresponding to Figure 4D.pdf]

Figure 4D

uncropped blots

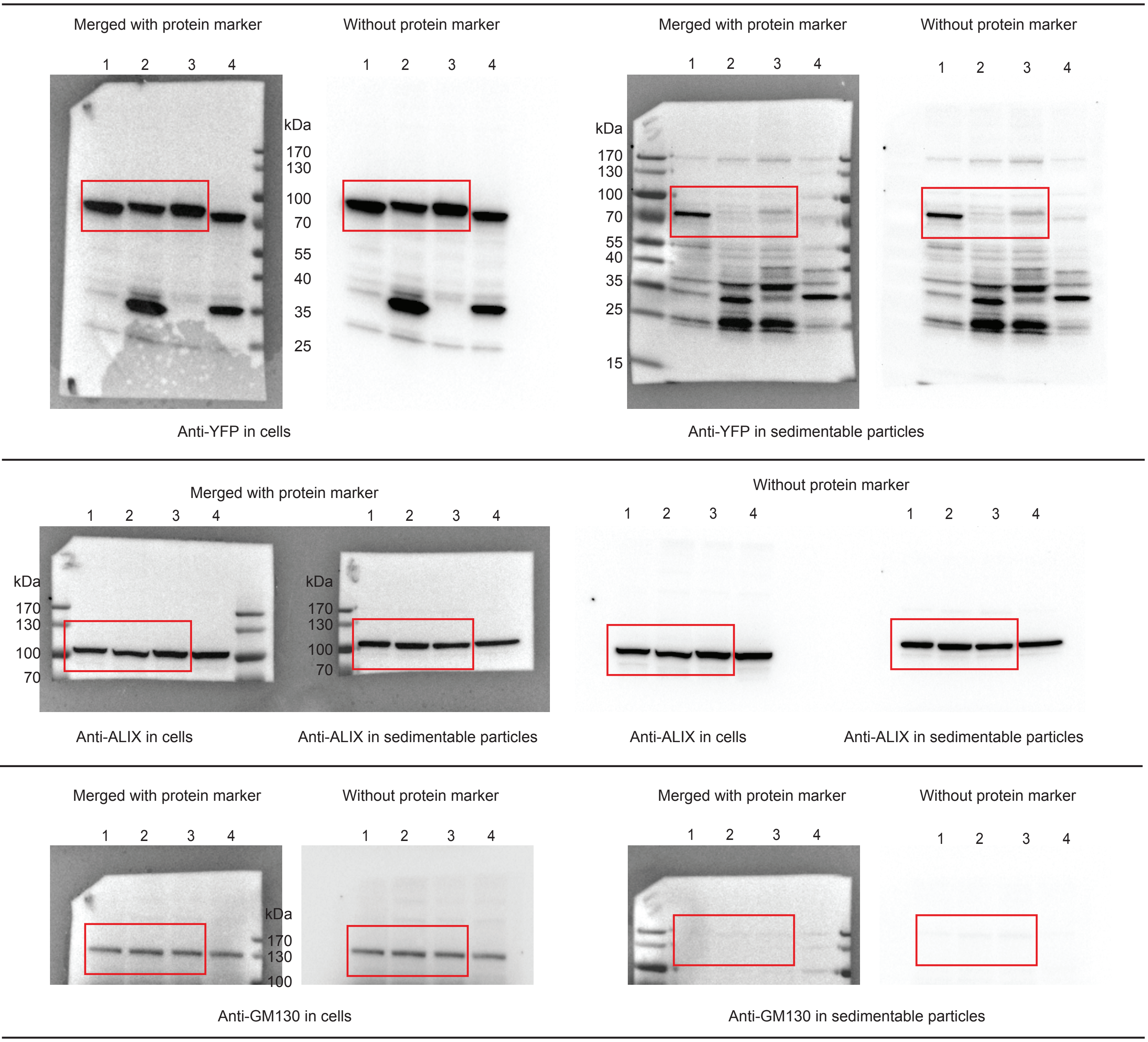

Lane 1: ΔYBX1, YFP-YBX1-WT,
Lane 2: ΔYBX1, YFP-YBX1-LCD-Y to S,
Lane 3: ΔYBX1, YFP-YBX1-LCD-RK to G,
Lane 4: ΔYBX1, YFP-YBX1-F85A.

Lines 1, 2, and 3 were used in the Figue 4D

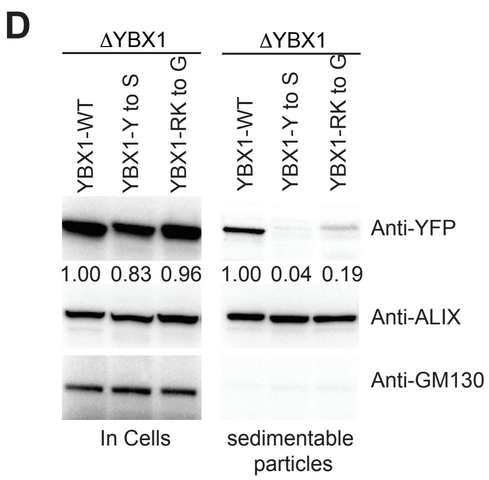

Figure 4D. IDR-driven YBX1 phase separation is required for YBX1 secretion in EVs.
