# Supplementary material for: Selective sorting of microRNAs into exosomes by phase-separated YBX1 condensates
Source: eLife. 2021 Nov 12;10:e71982. doi: 10.7554/eLife.71982 (PMC8612733; doi:10.7554/eLife.71982)
Supplement: Figure 4—source data 3. [file elife-71982-fig4-data3.zip › Figure 4-source data 3 for figure 4E/Uncropped Western blot images corresponding to Figure 4E.pdf]

Figure 4E

uncropped blots

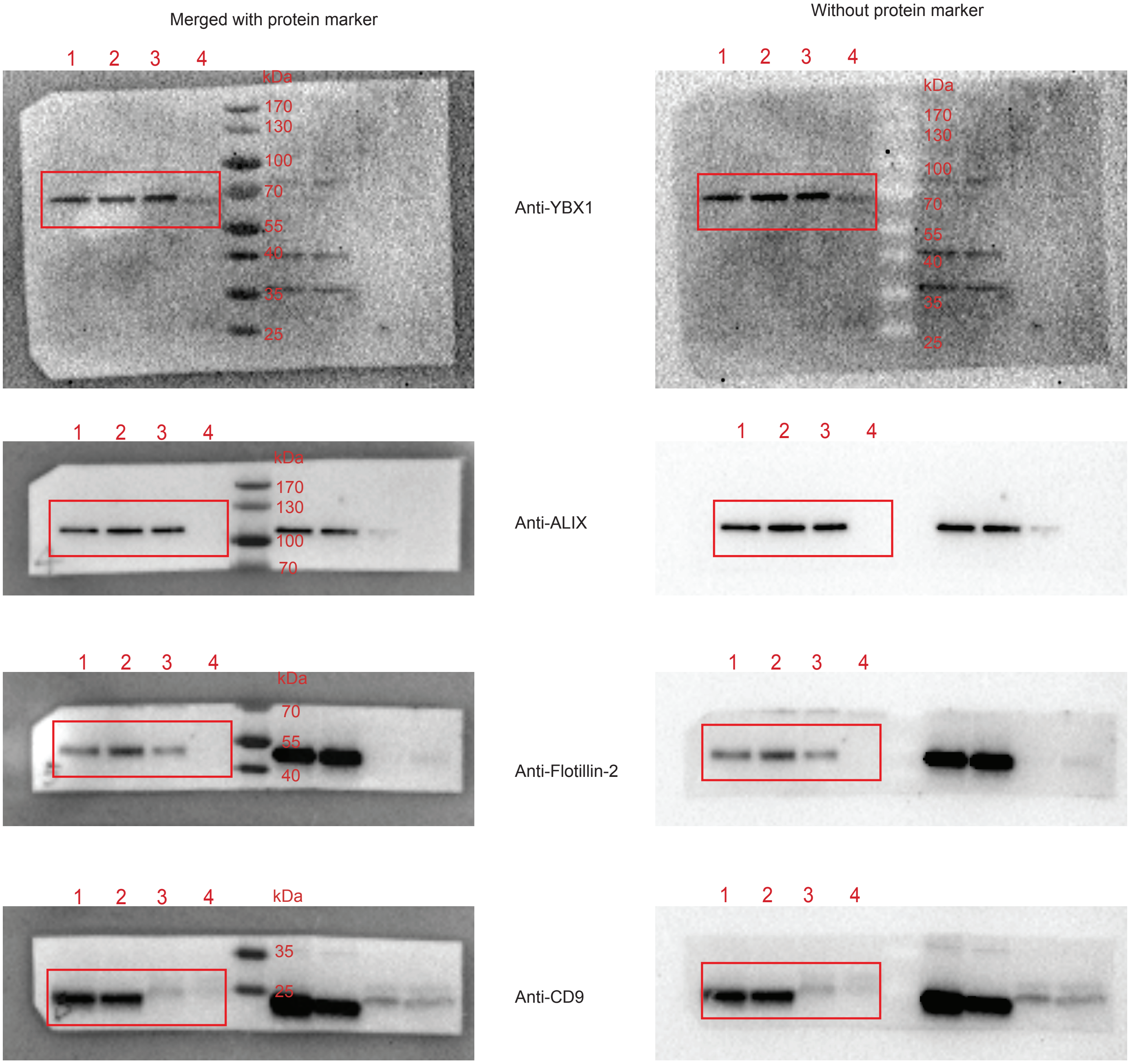

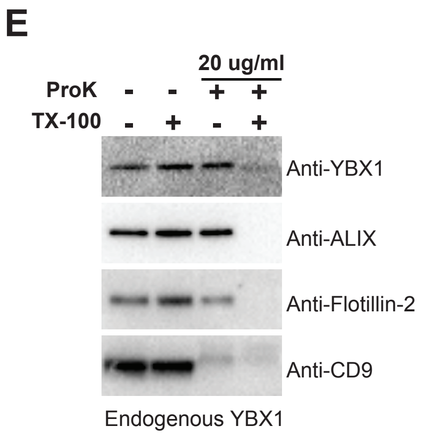

Lane 1: untreated,
Lane 2: treated with TX-100,
Lane 3: treated with ProK,
Lane 4: treated with both TX-100 and ProK

Lines 1, 2, 3 and 4 were used in the Figue 4E.

Figure 4E. Proteinase K protection assay on high-speed pellet fractions.
