# Supplementary material for: Selective sorting of microRNAs into exosomes by phase-separated YBX1 condensates
Source: eLife. 2021 Nov 12;10:e71982. doi: 10.7554/eLife.71982 (PMC8612733; doi:10.7554/eLife.71982)
Supplement: Figure 4—source data 4. [file elife-71982-fig4-data4.zip › Figure 4-source data 4 for Figure 4F/Uncropped Western blot images corresponding to Figure 4F.pdf]

Figure 4F

uncropped blots

Without protein marker

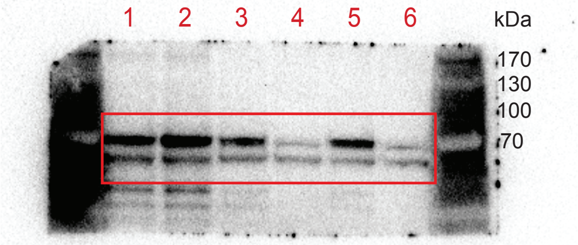

Anti-YBX1 in sedimentable particles

Merged with protein marker

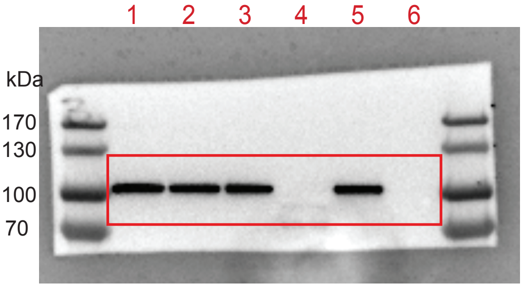

Anti-ALIX in sedimentable particles

without protein marker

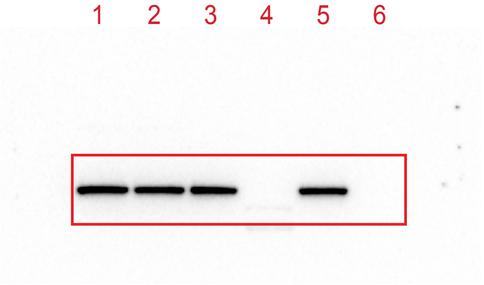

Merged with protein marker

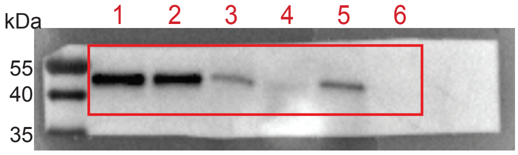

Anti-Flotillin-2 in sedimentable particles

Without protein marker

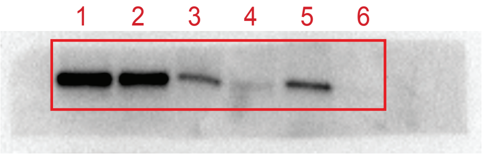

Merged with protein marker

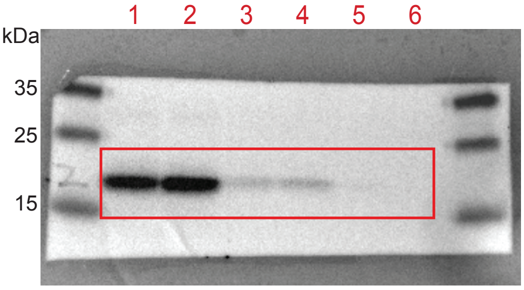

Anti-CD9 in sedimentable particles

Without protein marker

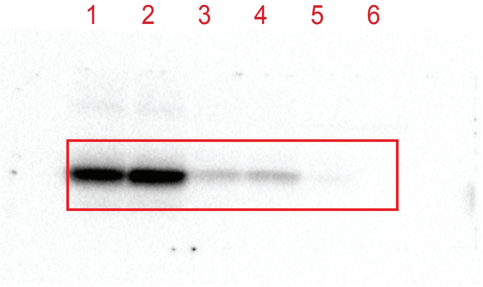

Lane 1: untreated,
Lane 2: treated with TX-100,
Lane 3: treated with 5 ug/ml ProK,
Lane 4: treated with both TX-100 and 5 ug/ml ProK,
Lane 5: treated with 20 ug/ml ProK,
Lane 6: treated with both TX-100 and 20 ug/ml ProK,

Lines 1, 2, 3, 4, 5 and 6 were used in the Figue 4F

F

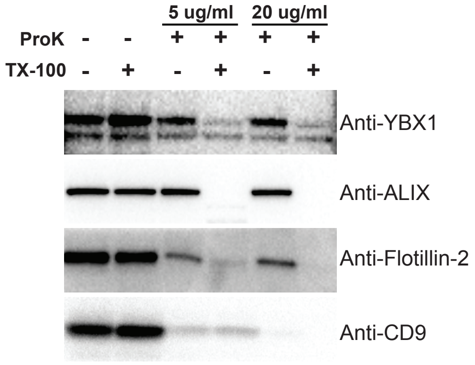

YFP-YBX1

Figure 4F. Proteinase K protection assay on high-speed pellet fractions from U2OS cells expressing YFP-YBX1.
