# Supplementary material for: Selective sorting of microRNAs into exosomes by phase-separated YBX1 condensates
Source: eLife. 2021 Nov 12;10:e71982. doi: 10.7554/eLife.71982 (PMC8612733; doi:10.7554/eLife.71982)
Supplement: Figure 4—source data 6. [file elife-71982-fig4-data6.zip › Figure 4-source data 6 for Figure 4K/Uncropped Western blot images corresponding to Figure 4K.pdf]

Figure 4K

uncropped blots

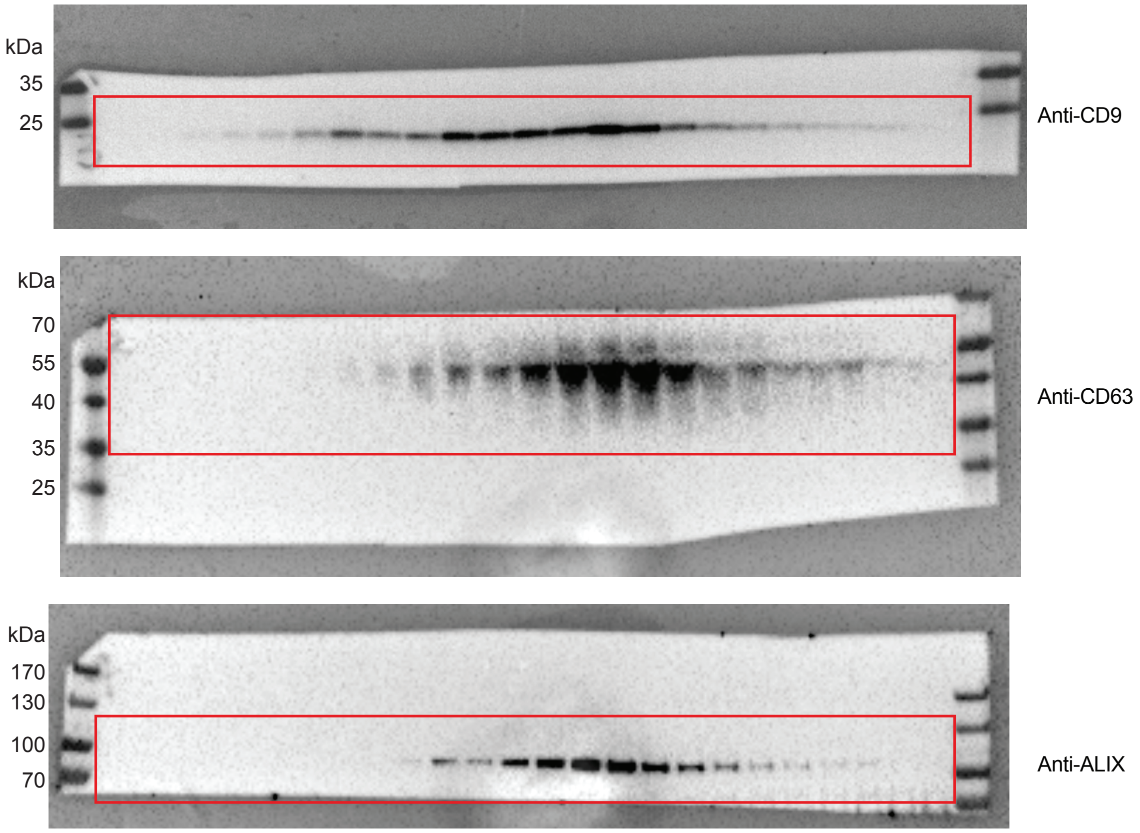

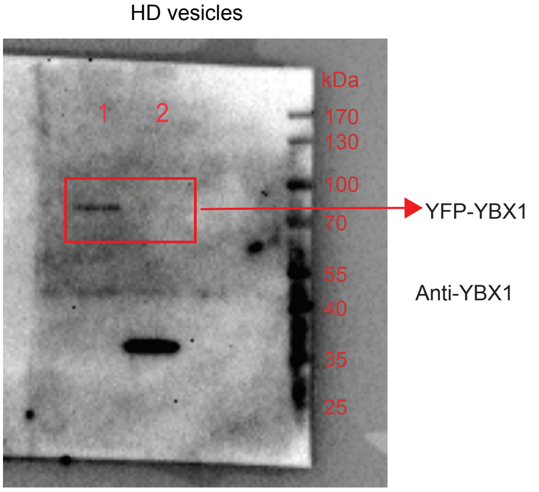

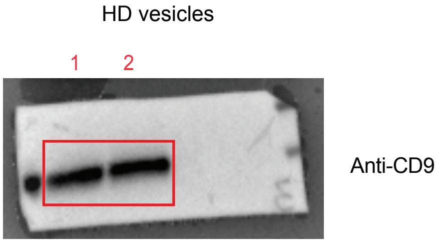

Lane 1: ΔYBX1, YFP-YBX1-WT,
Lane 2: ΔYBX1, YFP-YBX1-F85A.

Lane 1 and Lane 2 were used in Figure 4K (right panel).

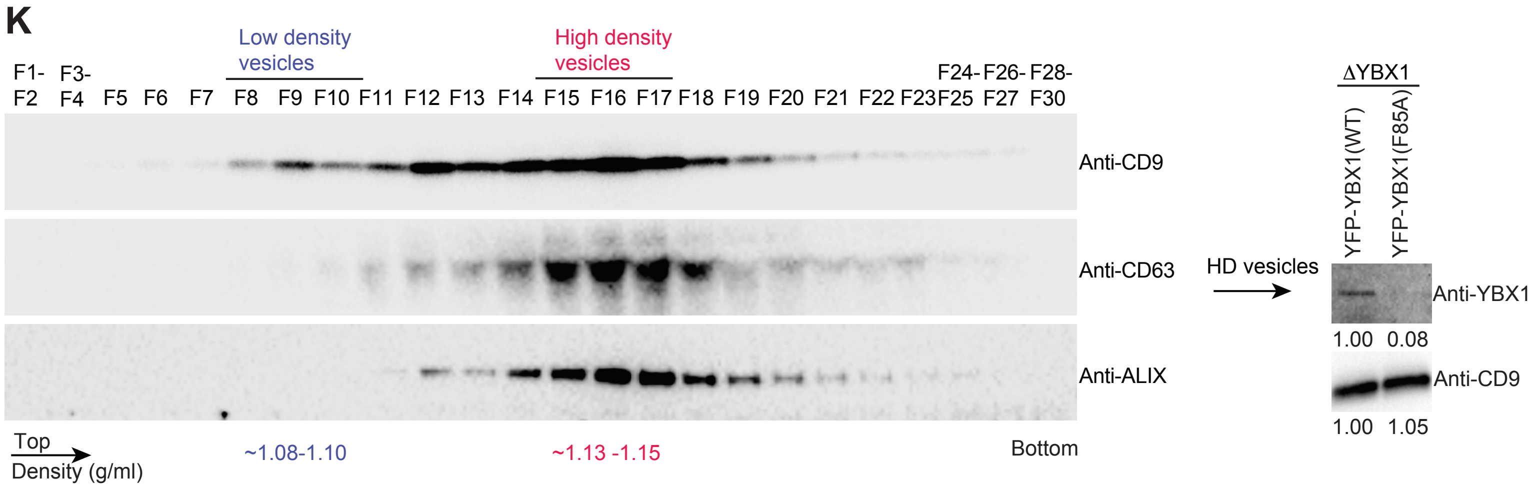

Figure 4K. Immunoblots across the iodixanol gradient for classical exosome markers CD9, CD63 and ALIX (the left panel) and YBX1 (the right panel).
