# Supplementary material for: Selective sorting of microRNAs into exosomes by phase-separated YBX1 condensates
Source: eLife. 2021 Nov 12;10:e71982. doi: 10.7554/eLife.71982 (PMC8612733; doi:10.7554/eLife.71982)
Supplement: Figure 5—source data 1. [file elife-71982-fig5-data1.zip › Figure 5-source data 1 for Figure 5C/Uncropped Western blot images corresponding to Figure 5C.pdf]

Figure 5C

uncropped blots

Merged with protein marker

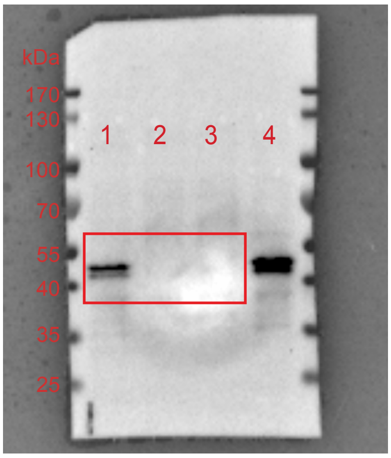

Anti-YBX1

Without protein marker

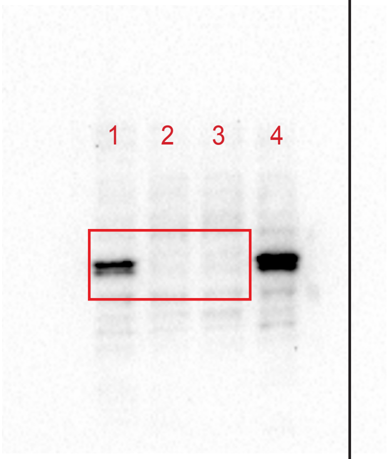

Anti-YBX1

Merged with protein marker

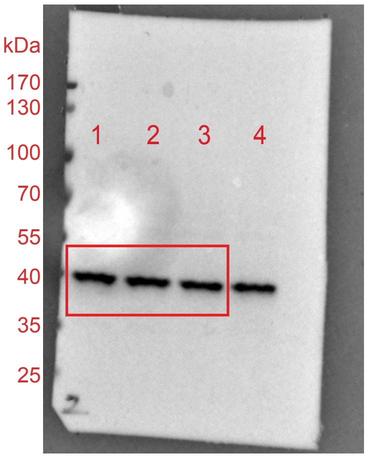

Anti-Actin

Without protein marker

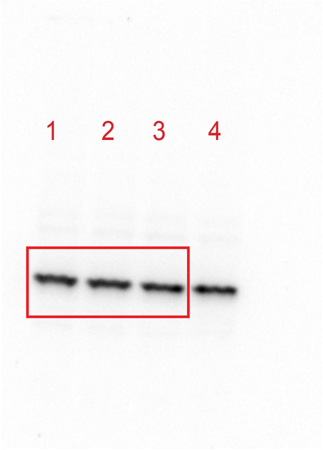

Anti-Actin

Lane 1: WT-1,
Lane 2: ΔYBX1-9,
Lane 3: ΔYBX1-41,
Lane 4: WT-2.

Lines 1, 2 and 3 were used in the Figue 5C.

C

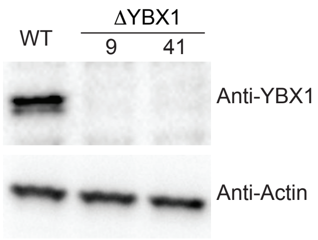

Figure 5C. Analysis of wild-type and CRISPR/Cas9 genome edited HEK293T clones by immunoblot for YBX1 (top) and actin (bottom).
