# Supplementary material for: Selective sorting of microRNAs into exosomes by phase-separated YBX1 condensates
Source: eLife. 2021 Nov 12;10:e71982. doi: 10.7554/eLife.71982 (PMC8612733; doi:10.7554/eLife.71982)
Supplement: Figure 7—source data 3. [file elife-71982-fig7-data3.zip › Figure 7-source data 3 for Figure 7G/Uncropped Western blot images corresponding to Figure 7G.pdf]

Figure 7G

uncropped blots

Merged with protein marker

Without protein marker

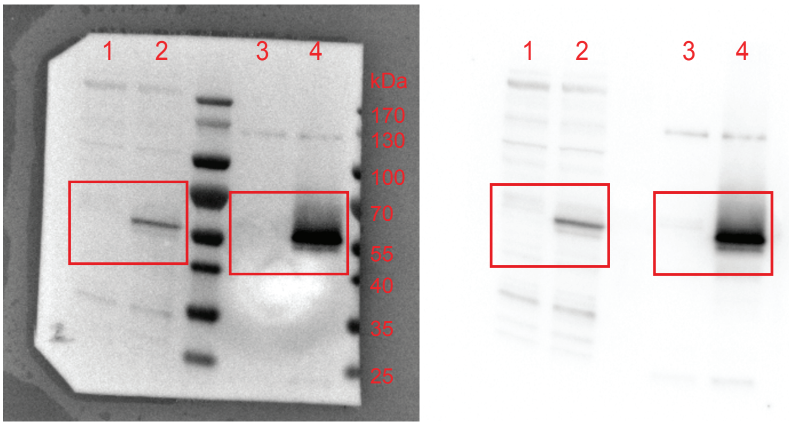

Lane 1: No tag, Input, anti-Flag
Lane 2: 3xFlag-YBX1, Input, anti-Flag
Lane 3: No tag, IP, anti-Flag
Lane 4: 3xFlag-YBX1, IP, anti-Flag

Anti-Flag

Merged with protein marker

Without protein marker

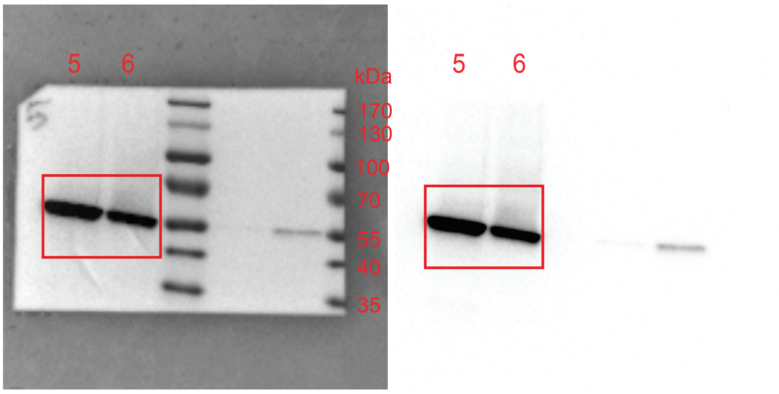

Anti-DDX6 for Input

Merged with protein marker

Without protein marker

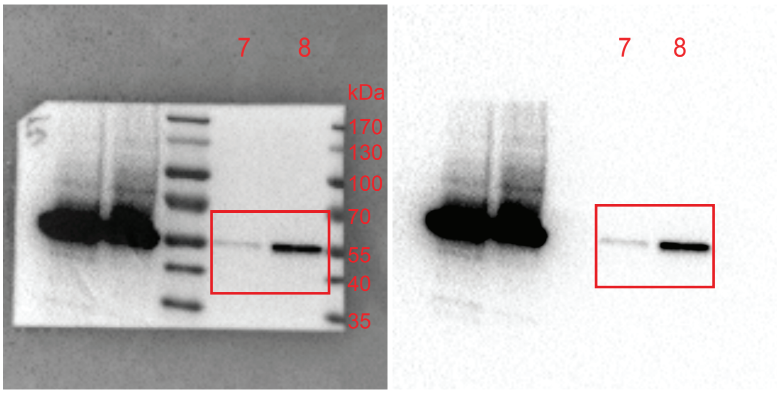

Anti-DDX6 for IP

Lane 5: No tag, Input, anti-DDX6
Lane 6: 3xFlag-YBX1, Input, anti-DDX6
Lane 7: No tag, IP, anti-DDX6
Lane 8: 3xFlag-YBX1, IP, anti-DDX6

Lane 1, 2, 3, 4, 5, 6, 7 and 8 were used in Figure 7G.

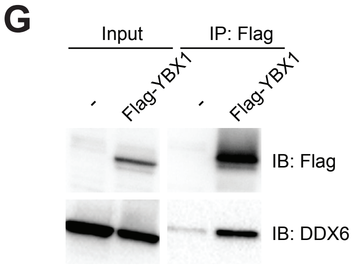

Figure 7G. Coimmunoprecipitation of DDX6 with YBX1.
