# Supplementary material for: Selective sorting of microRNAs into exosomes by phase-separated YBX1 condensates
Source: eLife. 2021 Nov 12;10:e71982. doi: 10.7554/eLife.71982 (PMC8612733; doi:10.7554/eLife.71982)
Supplement: Figure 7—source data 4. [file elife-71982-fig7-data4.zip › Figure 7-source data 4 for Figure 7H/Uncropped Western blot images corresponding to Figure 7H.pdf]

Figure 7H

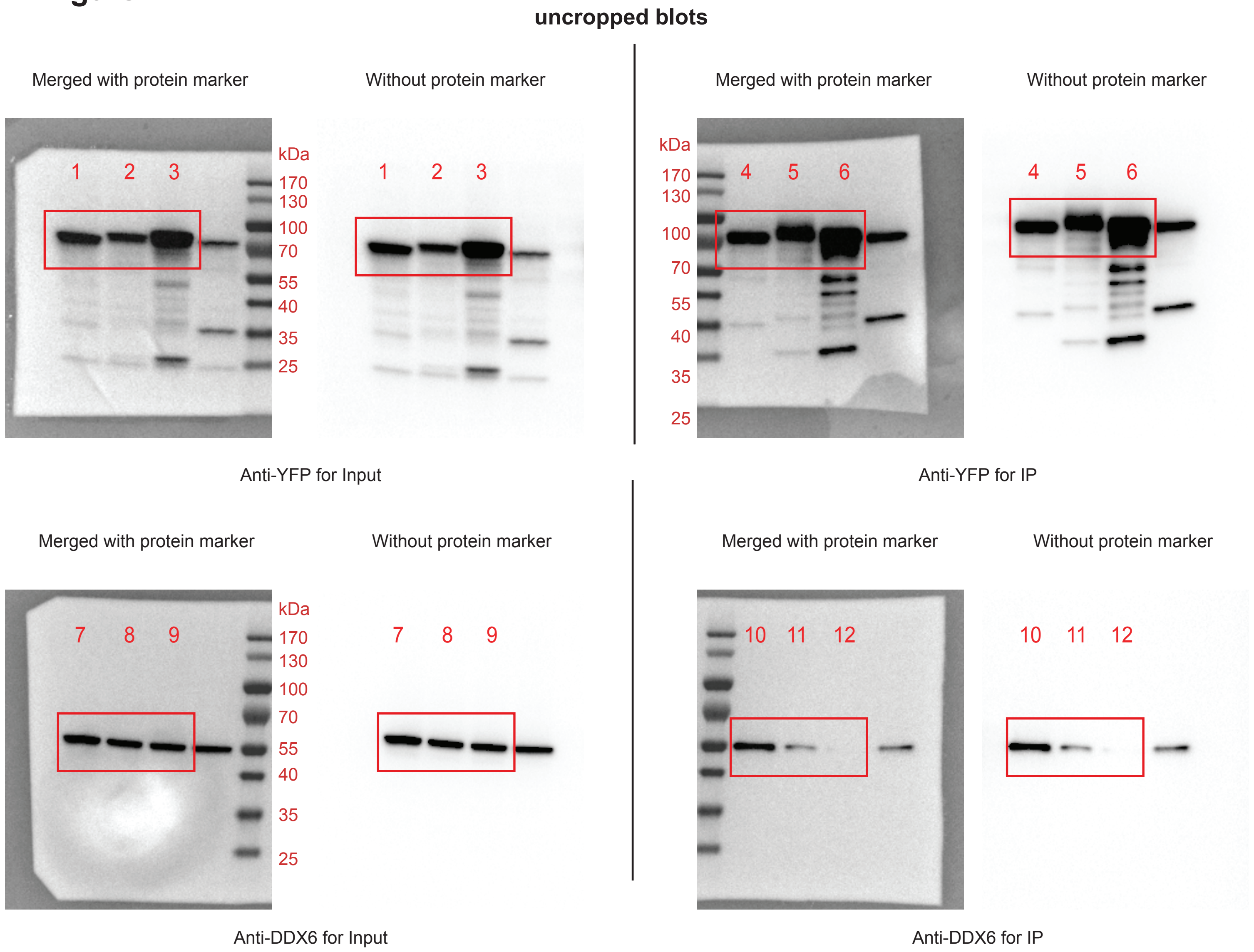

Lane 1: ΔYBX1, YFP-YBX1-WT, Input, anti-YFP
Lane 2: ΔYBX1, YFP-YBX1-LCD-Y to S, Input, anti-YFP
Lane 3: ΔYBX1, YFP-YBX1-LCD-RK to G, Input, anti-YFP
Lane 4: ΔYBX1, YFP-YBX1-WT, IP, anti-YFP
Lane 5: ΔYBX1, YFP-YBX1-LCD-Y to S, IP, anti-YFP
Lane 6: ΔYBX1, YFP-YBX1-LCD-RK to G, IP, anti-YFP

Lane 7: ΔYBX1, YFP-YBX1-WT, Input, anti-DDX6
Lane 8: ΔYBX1, YFP-YBX1-LCD-Y to S, Input, anti-DDX6
Lane 9: ΔYBX1, YFP-YBX1-LCD-RK to G, Input, anti-DDX6
Lane 10: ΔYBX1, YFP-YBX1-WT, IP, anti-DDX6
Lane 11: ΔYBX1, YFP-YBX1-LCD-Y to S, IP, anti-DDX6
Lane 12: ΔYBX1, YFP-YBX1-LCD-RK to G, IP, anti-DDX6

Lanes 1-12 were used in Fig 7H.

H

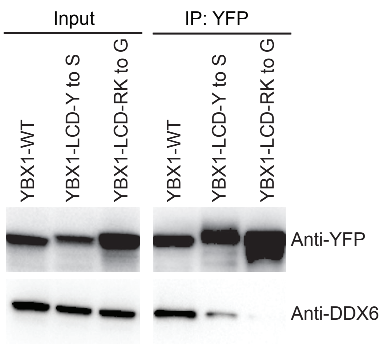

Figure 7H. Residues in YBX1-IDR that drive LLPS are required for its interaction with DDX6.
