# Supplementary material for: Selective sorting of microRNAs into exosomes by phase-separated YBX1 condensates
Source: eLife. 2021 Nov 12;10:e71982. doi: 10.7554/eLife.71982 (PMC8612733; doi:10.7554/eLife.71982)
Supplement: Figure 7—source data 5. [file elife-71982-fig7-data5.zip › Figure 7-source data 5 for Figure 7I/Uncropped Western blot images corresponding to Figure 7I.pdf]

Figure 7I

uncropped blots

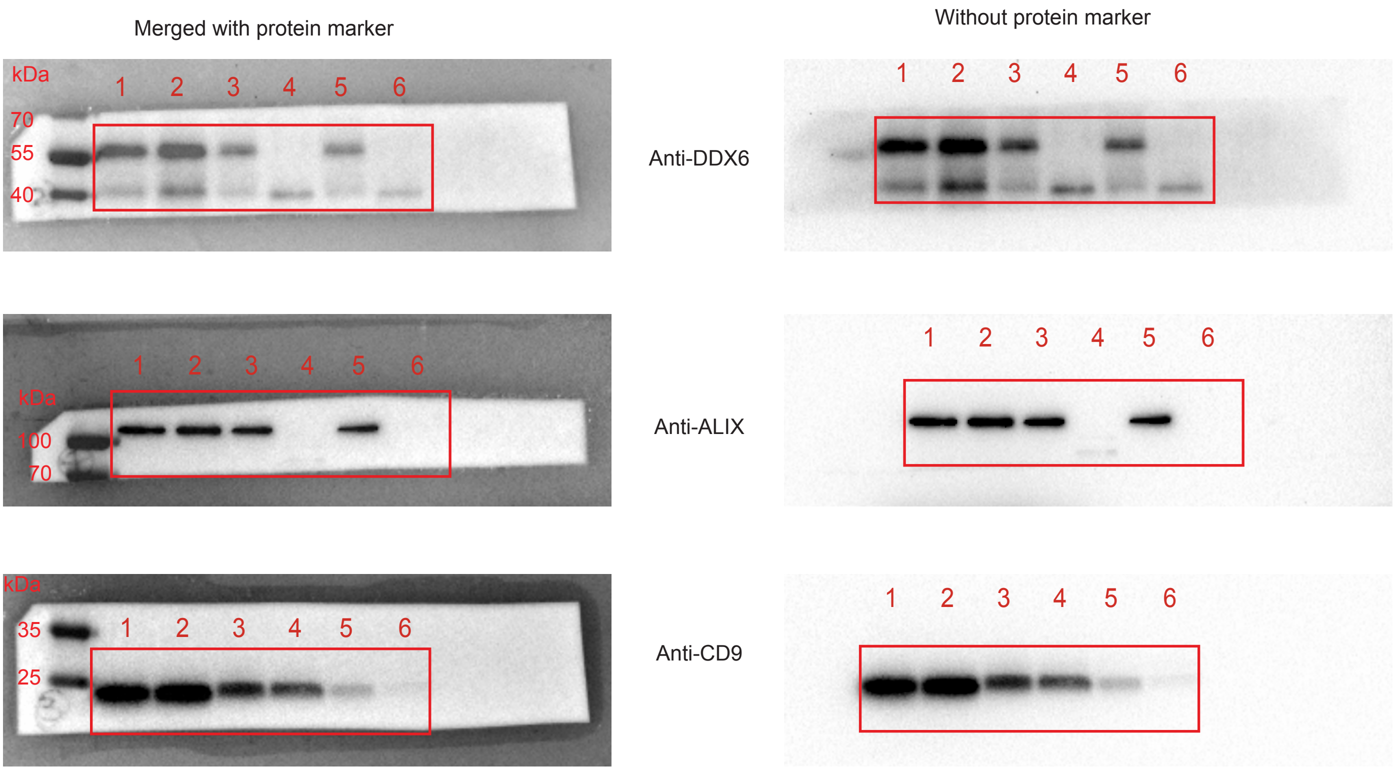

Lane 1: untreated,
Lane 2: treated with TX-100,
Lane 3: treated with 5 ug/ml ProK,
Lane 4: treated with both TX-100 and 5 ug/ml ProK,
Lane 5: treated with 20 ug/ml ProK,
Lane 6: treated with both TX-100 and 20 ug/ml ProK,

Lines 1, 2, 3, 4, 5 and 6 were used in the Figue 7I

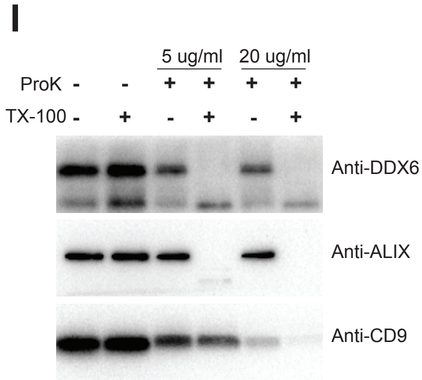

Figure 7I. Proteinase K protection assay for DDX6 using exosomes that were isolated by buoyant density flotation.
