# Supplementary material for: Selective sorting of microRNAs into exosomes by phase-separated YBX1 condensates
Source: eLife. 2021 Nov 12;10:e71982. doi: 10.7554/eLife.71982 (PMC8612733; doi:10.7554/eLife.71982)
Supplement: Figure 7—figure supplement 3—source data 1. [file elife-71982-fig7-figsupp3-data1.zip › Figure 7-supplement 3-source data 1/Uncropped Western blot images corresponding to Figure 7-supplement 3.pdf]

Figure 7-supplement 3B

uncropped blots

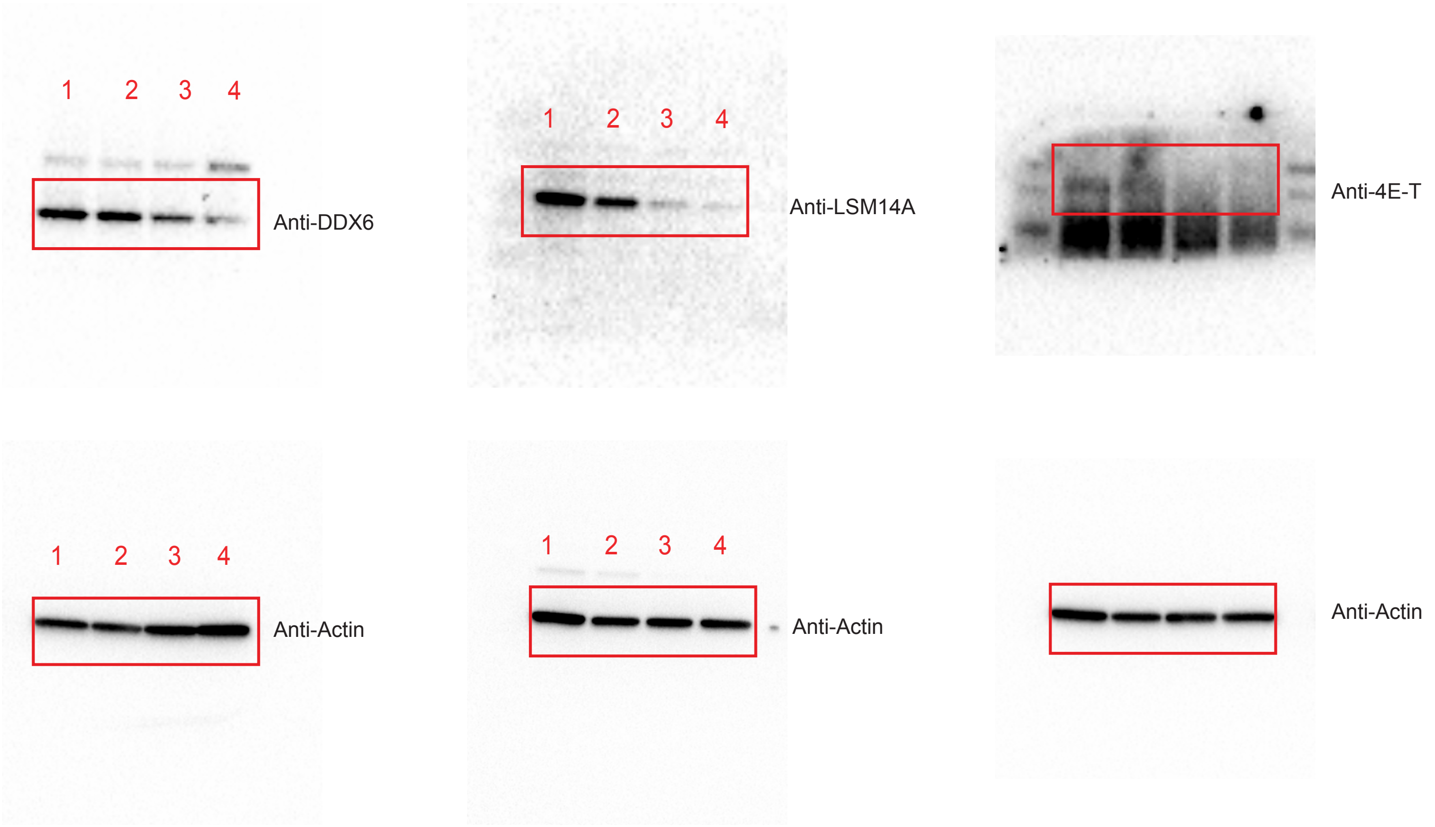

B

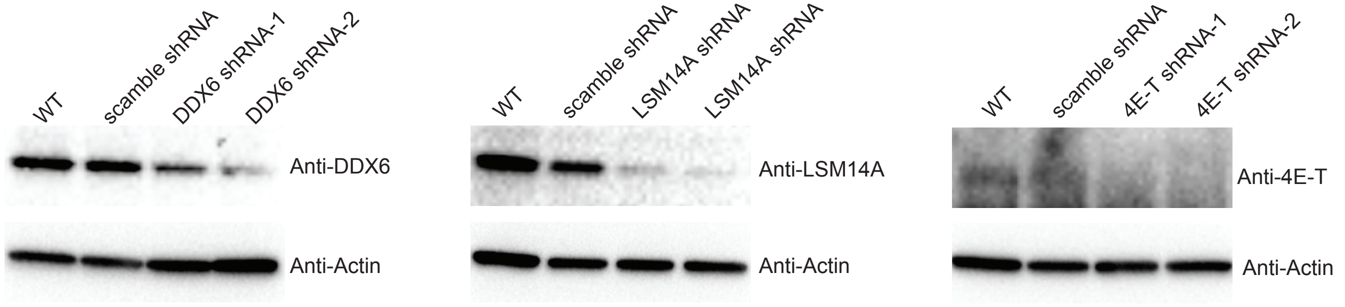

Figure 7-figure supplement 3. Effects of shRNA knockdown of selected P body proteins on miR-223 sorting into exosomes.
