# Supplementary material for: Selective sorting of microRNAs into exosomes by phase-separated YBX1 condensates
Source: eLife. 2021 Nov 12;10:e71982. doi: 10.7554/eLife.71982 (PMC8612733; doi:10.7554/eLife.71982)
Supplement: Figure 7—figure supplement 3—source data 2. [file elife-71982-fig7-figsupp3-data2.docx]

**Table 4. Oligo sequences used for shRNA cloning**

| shRNA name | Oligonucleotide sequence 5’-3’ |
| --- | --- |
| DDX6-shRNA-1  top strand | CCGGGAACATCGAAATCGTGTATTTCTCGAGAAATACACGATTTCGATGTTCTTTTTG |
| DDX6-shRNA-1  bottom strand | AATTCAAAAAGAACATCGAAATCGTGTATTTCTCGAGAAATACACGATTTCGATGTTC |
| DDX6-shRNA-2  top strand | CCGGGAGTATGACCACCACTATTAACTCGAGTTAATAGTGGTGGTCATACTCTTTTTG |
| DDX6-shRNA-2  bottom strand | AATTCAAAAAGAGTATGACCACCACTATTAACTCGAGTTAATAGTGGTGGTCATACTC |
| 4E-T-shRNA-1  top strand | CCGGGGATCACCGTCTTAGCGATAACTCGAGTTATCGCTAAGACGGTGATCCTTTTTG |
| 4E-T-shRNA-1  bottom strand | AATTCAAAAAGGATCACCGTCTTAGCGATAACTCGAGTTATCGCTAAGACGGTGATCC |
| 4E-T-shRNA-2  top strand | CCGGCCTAAGGGTTCTGAGGAAATACTCGAGTATTTCCTCAGAACCCTTAGGTTTTTG |
| 4E-T-shRNA-2  bottom strand | AATTCAAAAACCTAAGGGTTCTGAGGAAATACTCGAGTATTTCCTCAGAACCCTTAGG |
| LSM14A-shRNA-1  top strand | CCGGTTCCGTGGGAGTGACATTAAACTCGAGTTTAATGTCACTCCCACGGAATTTTTG |
| LSM14A-shRNA-1 bottom strand | AATTCAAAAATTCCGTGGGAGTGACATTAAACTCGAGTTTAATGTCACTCCCACGGAA |
